# T cell receptor alpha variable 12‐2 bias in the immunodominant response to Yellow fever virus

**DOI:** 10.1002/eji.201747082

**Published:** 2017-12-11

**Authors:** Amandine Bovay, Vincent Zoete, Garry Dolton, Anna M. Bulek, David K. Cole, Pierre J. Rizkallah, Anna Fuller, Konrad Beck, Olivier Michielin, Daniel E. Speiser, Andrew K. Sewell, Silvia A. Fuertes Marraco

**Affiliations:** ^1^ Department of Oncology Lausanne University Hospital (CHUV) Epalinges Switzerland; ^2^ SIB Swiss Institute of Bioinformatics Molecular Modeling Group Lausanne Switzerland; ^3^ Division of Infection and Immunity and Systems Immunity Research Institute Cardiff University School of Medicine Heath Park Cardiff UK; ^4^ Cardiff University School of Dentistry Heath Park Cardiff UK

**Keywords:** Antigen recognition, Germline, T cell receptor Alpha Variable (TRAV)‐12‐2, T cell receptor bias, Yellow Fever virus

## Abstract

The repertoire of human αβ T‐cell receptors (TCRs) is generated via somatic recombination of germline gene segments. Despite this enormous variation, certain epitopes can be immunodominant, associated with high frequencies of antigen‐specific T cells and/or exhibit bias toward a TCR gene segment. Here, we studied the TCR repertoire of the HLA‐A*0201‐restricted epitope LLWNGPMAV (hereafter, A2/LLW) from Yellow Fever virus, which generates an immunodominant CD8^+^ T cell response to the highly effective YF‐17D vaccine. We discover that these A2/LLW‐specific CD8^+^ T cells are highly biased for the TCR α chain TRAV12‐2. This bias is already present in A2/LLW‐specific naïve T cells before vaccination with YF‐17D. Using CD8^+^ T cell clones, we show that TRAV12‐2 does not confer a functional advantage on a per cell basis. Molecular modeling indicated that the germline‐encoded complementarity determining region (CDR) 1α loop of TRAV12‐2 critically contributes to A2/LLW binding, in contrast to the conventional dominant dependence on somatically rearranged CDR3 loops. This germline component of antigen recognition may explain the unusually high precursor frequency, prevalence and immunodominance of T‐cell responses specific for the A2/LLW epitope.

## Introduction

Human αβ TCRs are heterodimeric proteins composed of an α‐ and β‐chain, somatically rearranged during T cell development from a selection of 176 variables (V), diversity (D), joining (J), and constant (C) genes 1. The specificity of peptide‐MHC (pMHC) recognition is conferred by the six highly flexible complementarity‐determining region (CDR) loops that make up the antigen‐binding site of the TCR. The CDR1 and CDR2 sequences are entirely encoded within the Variable genes for each α‐ and β‐chain (T cell Receptor Alpha Variable, TRAV, and T cell Receptor Beta Variable genes, TRBV, respectively). CDR1 and CDR2 are therefore entirely germline‐encoded. In contrast, the CDR3 loops, which generally make extensive contacts with the antigenic peptide 2, 3, are encoded by the V(D)J joints and thus hypervariable. The general consensus is that the somatic hypervariability of the CDR3 loops contributes most to the broad range of TCR specificities. However, with more atomic structures of TCR‐pMHC complexes, it is becoming evident that the germline CDR1α loop can sometimes also contact peptide residues, and in some cases dominate the contact with the peptide 4, 5, 6.

Antigen‐specific CD8^+^ T cells can be biased for certain TRAV or TRBV segments or feature “public TCRs” shared across the human population. In addition, TCR bias has been observed in infection, autoimmunity, and alloreactivity, with many examples reviewed by Turner et al. 7. The reasons behind the sharing of particular TCR segments are not yet fully understood but may have critical implications for the understanding and induction of optimal, protective antigen‐specific T cell responses.

Recently, we described the remarkable decade‐long persistence of human stem cell‐like memory (SCM) CD8^+^ T cells specific for the HLA‐A*0201‐restricted Yellow Fever virus (YFV) Non‐Structural protein 4b^214‐222^ epitope (sequence LLWNGPMAV; hereafter, A2/LLW) in the context of YF‐17D vaccination 8. Several studies have shown that this A2/LLW epitope is highly dominant and prevalent amongst YF‐17D vaccinees 9, 10, 11. At the peak of the T cell response, up to 25% of the peripheral CD8^+^ T cells can be specific for A2/LLW 9. In our hands, A2/LLW‐specific CD8^+^ T cells could be detected in 38/41 HLA‐A*0201 positive individuals after vaccination with YF‐17D (>90% prevalence) 8. Interestingly, we also revealed that naïve A2/LLW‐specific CD8^+^ T cells were readily detectable in 3 out of 10 unvaccinated donors 8. The reasons behind this unusual high frequency and prevalence of A2/LLW‐specific CD8^+^ T cells warranted further investigation. Here, we show that A2/LLW‐specific TCRs are highly biased for the TCRα chain germline segment TRAV12‐2. This finding is in common with another human specificity for which there is an extraordinarily high frequency of naïve T cells: the HLA‐A*0201‐restricted epitope ELAGIGILTV (heteroclitic analog to EAAGIGILTV from Melan‐A, and hereafter, A2/ELA) 12, 13. Pertinently, A2/ELA‐specific CD8^+^ T cells are also known to be biased for TRAV12‐2 and the germline‐encoded CDR1α loop in an A2/ELA‐specific TCR featuring TRAV12‐2 (MEL5 TCR) makes major contributions to antigen recognition, thereby providing a so called “innate‐like” binding of the peptide 13, 14, 15, 16, 17, 18. We performed functional and structural studies to further investigate the TRAV12‐2 bias in A2/LLW‐specific CD8^+^ T cells.

## Results

### A2/LLW‐specific CD8^+^ T cells are biased for the segment TRAV12‐2, before and after vaccination

**Figure 1:**
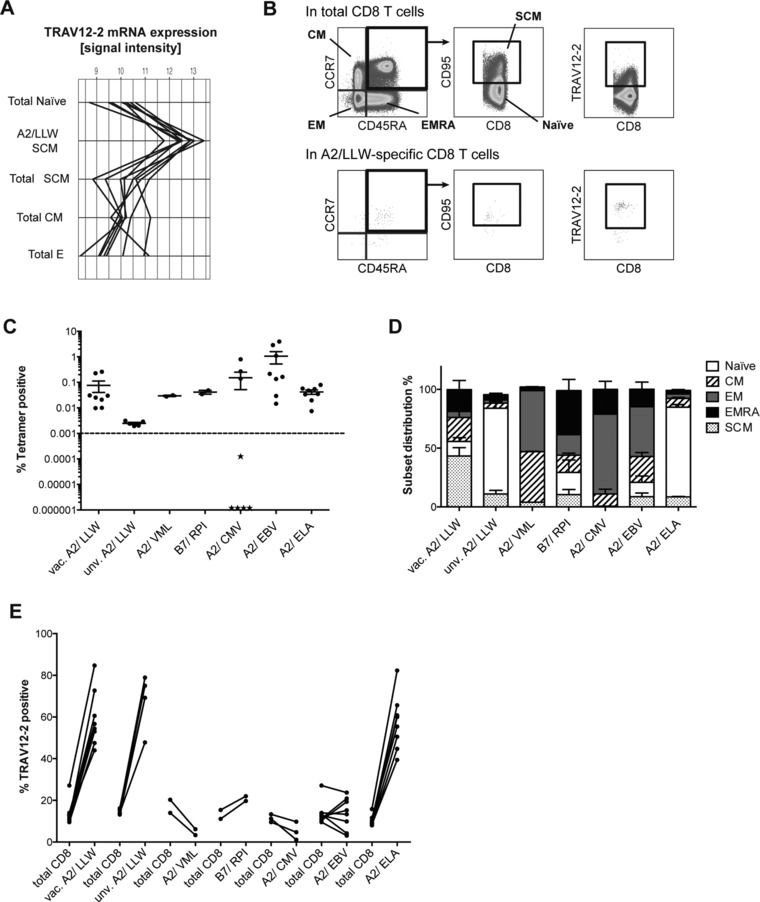
A2/LLW‐specific CD8^+^ T cells are strongly biased for TRAV12‐2 similarly to A2/ELA‐specific CD8^+^ T cells. (A) TRAV12‐2 mRNA expression in A2/LLW‐specific stem cell‐like (SCM) CD8^+^ T cells compared to reference differentiation subsets in total CD8^+^ T cells (*N* = 8 YF‐17D vaccinees), including: Naïve, SCM, central memory (CM) and effectors (E). Samples were isolated from PBMCs by FACS and total RNA analyzed by microarray. (B) Representative gating strategy for the flow cytometry analysis of CD8^+^ T cell subsets in total or tetramer positive populations and TRAV12‐2 expression therein. EM: effector memory; EMRA: effector memory CD45RA^+^. (C) Frequencies (%) of various antigen specificities amongst circulating CD8^+^ T cells (mean and SEM), including A2/LLW in YF‐17D vaccinees (*N* = 8) and unvaccinated individuals (*N* = 5), A2/VML (*N* = 2) and B7/RPI (*N* = 2) in YF‐17D vaccinees, as well as A2/CMV (*N* = 8; stars represent CMV‐seronegative donors = 5/8), A2/EBV (*N* = 8) and A2/ELA (*N* = 8). Data are representative of two independent experiments. (D) Subset distribution of antigen‐specific CD8^+^ T cell populations (mean and SEM). (E) Subject‐paired comparison of TRAV12‐2 expression between antigen‐specific and total CD8^+^ T cells (“vac.” = YF‐17D vaccinee; “unv.” = unvaccinated with YF‐17D).

Initially, genome‐wide analysis of A2/LLW‐specific CD8^+^ SCM and various differentiation subsets in total CD8^+^ T cells 8, 19 revealed that the most prominent feature was the highly significant enrichment of the TRAV12‐2 in A2/LLW‐specific CD8^+^ T cells (Fig. 1A). We next investigated this TRAV12‐2 enrichment in A2/LLW‐specific CD8^+^ T cells from eight YF‐17D vaccinees at the protein level (Figs. 1B and E). We also compared A2/LLW‐specific CD8^+^ T cells in these vaccinees to those in unvaccinated donors in order to address whether vaccination induced the observed TRAV12‐2 bias (Fig. 1E). In addition, we analyzed healthy donors for other antigen specificities such as A2/ELA, which is known to be biased for TRAV12‐2 13, and other viral antigen specificities that are not known to exhibit such bias: the HLA‐A*0201‐restricted epitopes BMFL1 from Epstein Barr Virus (EBV) and pp65 from cytomegalovirus (CMV) as well as two other YF epitopes that were detectable in two YF‐17D vaccinees (the HLA‐A*0201‐restricted VMLFILAGL from NS4a protein, A2/VML, and HLA‐B*07‐restricted RPIDDRFGL from NS5 protein, B7/RPI). In accordance with our previous study 8, we found that vaccinees had easily detectable and largely differentiated A2/LLW‐specific CD8^+^ T cells (Figs. 1B to D), while unvaccinated donors showed lower but detectable frequencies of naïve A2/LLW‐specific CD8^+^ T cells (i.e. above 0.001%) (Figs. 1C and D). As expected, naïve A2/ELA‐specific CD8^+^ T cells were also detectable in healthy donors, while the other viral antigen specificities were variably detected amongst donors and displayed differentiated phenotypes (Figs. 1C and D). The TRAV12‐2 segment was used by the majority (median 55.5%) of A2/LLW‐specific CD8^+^ T cells (Fig. 1E), in contrast to total CD8^+^ T cells (median 12.5%). The TRAV12‐2 bias reached a similar extent as in Melan‐A‐specific CD8^+^ T cells from healthy donors (median 57.7%), in contrast to the absence of bias in the other specificities (other two YF‐17D epitopes and CMV‐ and EBV‐specific epitopes) (Fig. 1E). Interestingly, we found that the TRAV12‐2 bias was already evident in naïve A2/LLW‐specific CD8^+^ T cells, prior to vaccination (median 69.2%).

### Despite the TRAV12‐2 bias, A2/LLW‐specific TCRs are mostly unique and public sequences infrequent

**Figure 2:**
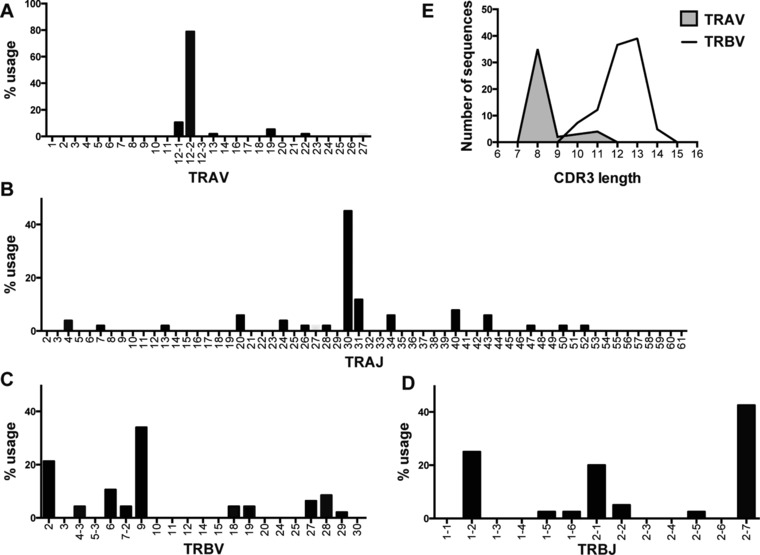
TCR repertoire analysis of A2/LLW‐specific CD8^+^ T cell clones generated from four vaccinated donors. Total RNA was isolated from 57 A2/LLW‐specific CD8^+^ T cell clones, cDNA prepared, analyzed by PCR with primers specific for each TRAV and TRBV gene segment, and sequenced. (A) TRAV gene usage. (B) TRAJ gene usage. (C) TRBV gene usage. (D) TRBJ gene usage. (E) CDR3 length distribution according to IMGT definition.

We generated and analyzed 57 A2/LLW‐specific CD8^+^ T cell clones derived from four different YF‐17D vaccinees. As shown in Fig. 2A, the Vα gene segments were predominated by TRAV12‐2, with 45 of 57 clones positive for TRAV12‐2 (78.9%). The TRAJs were relatively more diverse, using 15 of the 61 TRAJ human genes, yet consisting predominantly of the TRAJ30 (45.1%) (Fig. 2B). In contrast, the Vβ repertoire was highly heterogeneous, with 10 different Vβ segments used, although a moderate bias for some TRBV genes was noted: TRBV9 was used by 16 clones and TRBV2 used by 10 clones (Fig. 2C). There was no evident TRBJ bias (Fig. 2D). In addition, TRAV12‐2 CDR3 length consisted predominantly of 8 amino acids whereas CDR3β sequences showed a broader distribution (Fig. 2E). Most TCRs were unique clonotypes (Supporting Information Table 1), with no conserved motif in the CDR3 loop observed. We identified two public TRAV sequences: “CAVTDDKIIFG” was shared by all four donors and “CAVGDDKIIFG” by three out of four donors.

### On a per cell basis, TRAV12‐2 does not confer functional advantages to A2/LLW‐specific CD8^+^ T cells

**Figure 3:**
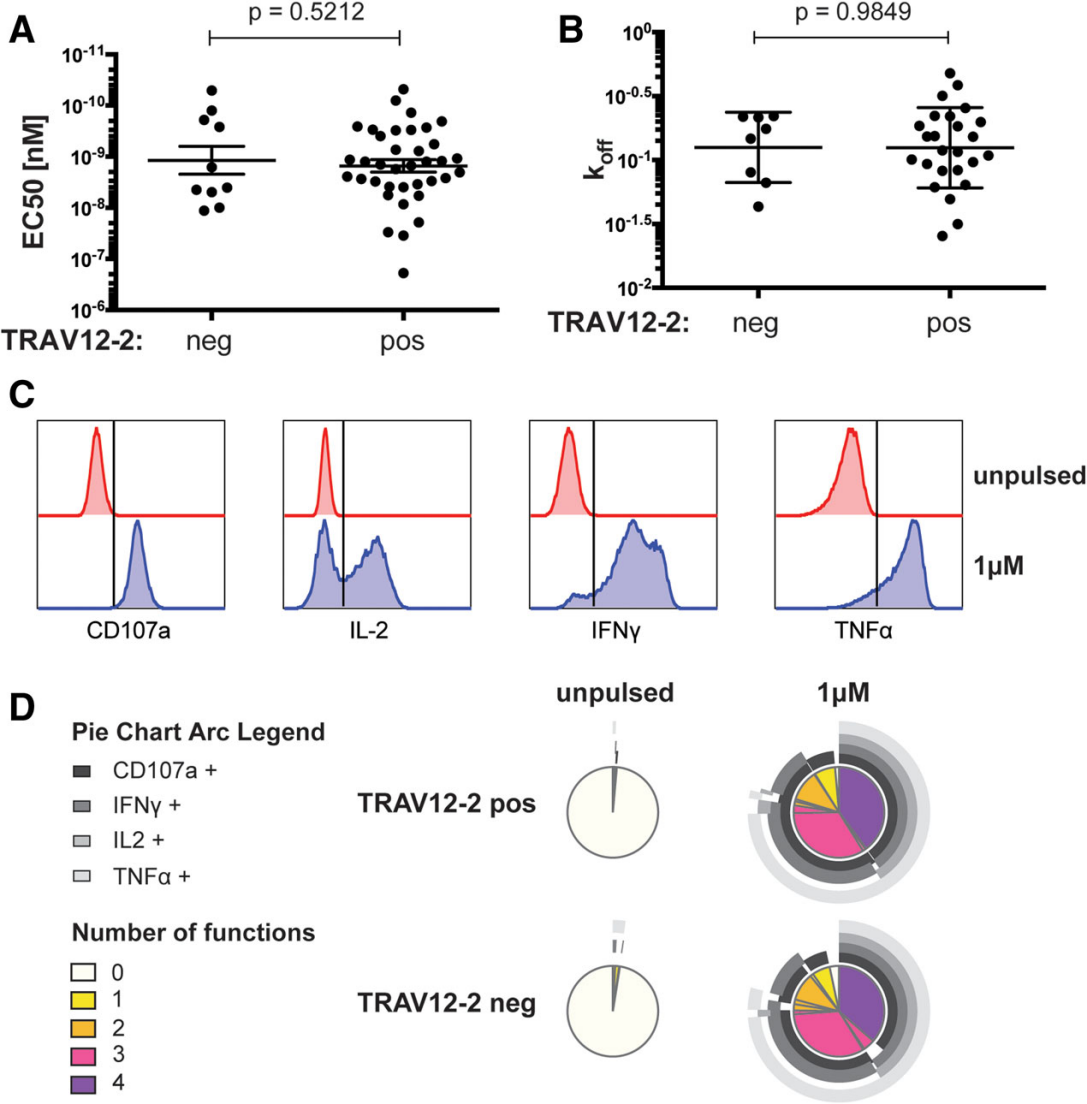
TRAV12‐2 expression does not confer a functional advantage. Functional properties of A2/LLW‐specific CD8^+^ T cell clones were assessed by various methods. (A) Killing capacity (51‐chromium release assay) with LLW peptide titration in A2/LLW‐specific CD8^+^ T cell clones (TRAV12‐2 positive *N* = 37, TRAV12‐2 negative *N* = 10). Data are representative of two independent experiments (mean and SEM; *t*‐test *p* value). (B) Monomeric dissociation constant (K_off_) rates measured in CD8^+^ T cell clones (TRAV12‐2 positive *N* = 25, TRAV12 negative = 8) using NTAmers (mean and SD; *t*‐test P value). (C and D) Intracellular cytokine staining of CD8^+^ T cell clones (TRAV12‐2 positive *N* = 11, TRAV12‐2 negative *N* = 6) following LLW peptide stimulation for 4 h, showing representative flow cytometry gating strategy in C. Data are representative of two independent experiments.

One hypothesis could be that TCRs with TRAV12‐2 mediate increased T cell function. Analysis of various functional properties in A2/LLW‐specific CD8^+^ T cell clones showed that TRAV12‐2‐positive clones did not differ from TRAV12‐2‐negative clones, whether in killing capacity (EC_50_ in Fig. 3A), TCR avidity (K_off_ in Fig. 3B) or degranulation and secretion of IFN‐γ, TNF‐α, and IL‐2 after 4‐hours peptide stimulation (Fig. 3C and D). Altogether, expression of TRAV12‐2 did not confer a particular functional advantage in A2/LLW‐specific CD8^+^ T cell clones.

### The LLW peptide binds with high stability to HLA‐A*0201

**Figure 4:**
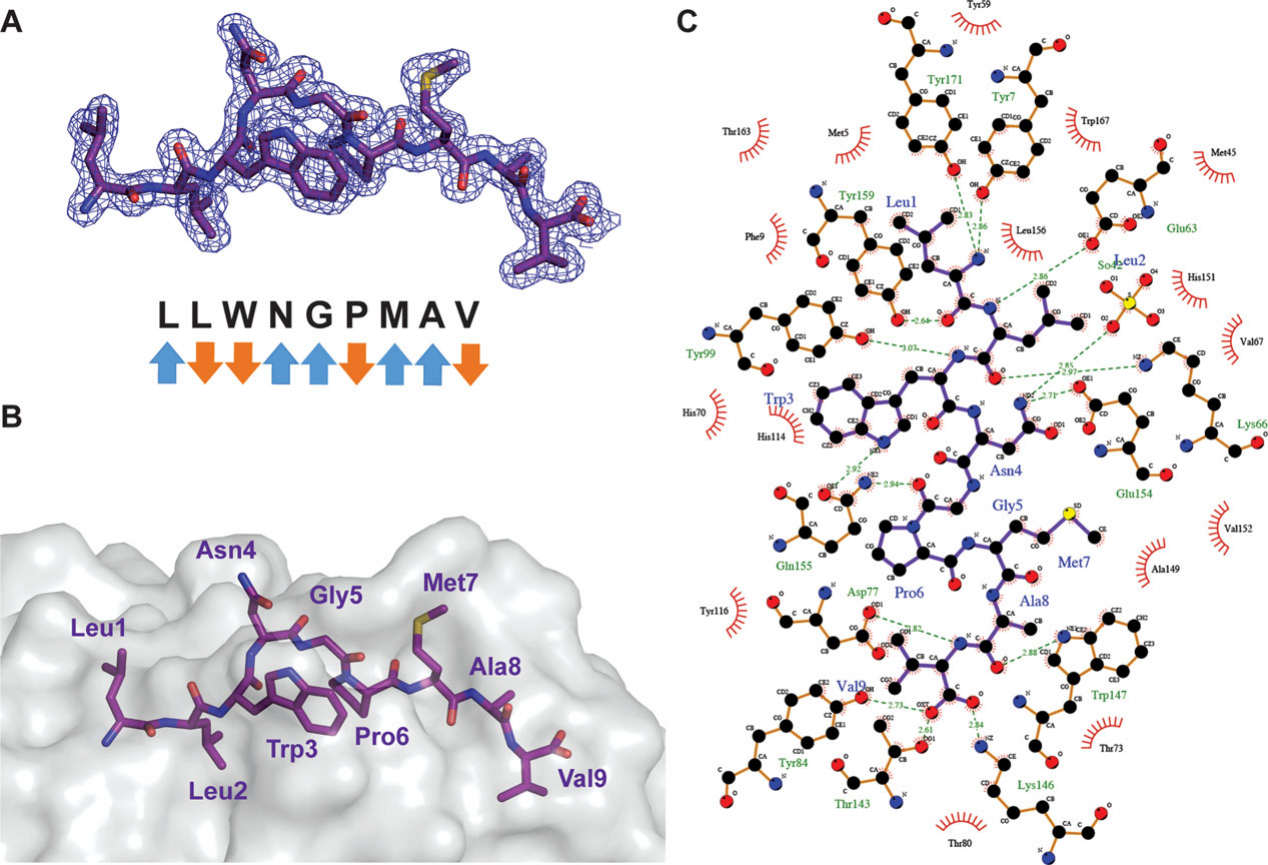
LLW peptide binds stably to HLA‐A*0201. (A) Electron density at 1σ contour level around the peptide of the A2/LLW complex showing the overall conformation of the peptide. Blue arrows (TCR‐exposed) and orange arrows (MHC‐buried) indicate direction of amino acid side chains. (B) Surface and stick representation of HLA‐A*0201 and peptide residues, respectively. (C) LIGPLOT schematic diagram showing the various interactions of the LLW peptide with HLA‐A*0201. Purple lines are peptide covalent bonds, orange lines are HLA‐A2 covalent bonds, dotted green lines are polar/H‐bond contacts, and open red arcs indicate a protein atom in a non‐polar contact.

The TRAV12‐2 bias in A2/LLW‐specific TCRs is reminiscent of the TRAV12‐2 bias observed in A2/ELA‐specific CD8^+^ T cells. In the A2/ELA‐specific MEL5 TCR structure, the germline‐encoded CDR1α loop makes important interactions with the ELA peptide in complex with HLA‐A*0201 providing an explanation for the preferential TRAV12‐2 usage and high frequency of this specificity 5, 14, 15, 16, 20, 21, 22. In order to determine the structural characteristics that govern the TCR recognition of the LLW peptide, we intended to solve the crystal structure of a TRAV12‐2 positive TCR specific for A2/LLW in complex with its cognate pMHC (A2/LLW). Unfortunately, despite several attempts with different TCRs, we were unable to refold a functional A2/LLW‐specific TRAV12‐2 positive TCR. We were able to solve the atomic structure of the A2/LLW pMHC complex at 1.59Å resolution. Electron density around the peptide was unambiguous (Fig. 4A). The general features of the binding are similar to those observed in other peptide‐HLA‐A*0201 complexes: the nonamer adopts a conformation with central bulge between residues at position 4 and 6 where the side chains are protruding toward the TCR (Fig. 4B), while the peptide termini provide binding into the HLA binding pockets with many specific interactions (Figs. 4A and C).

**Figure 5:**
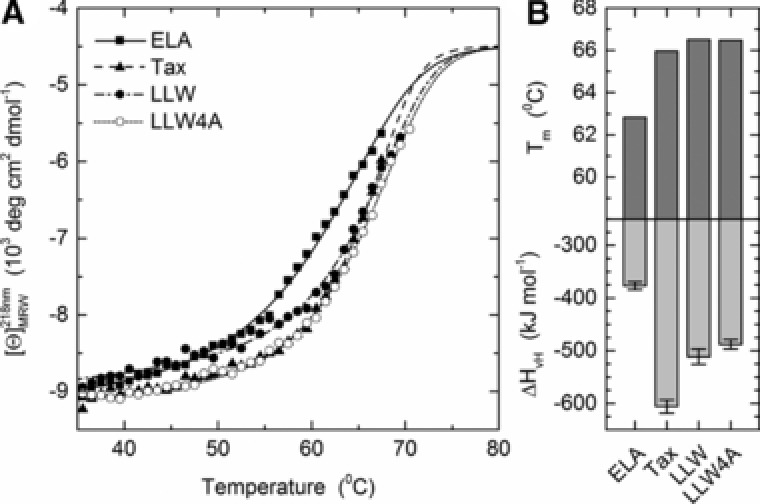
Thermal stability of pMHCs. (A) Temperature stability of the HLA‐A2*0201 molecules with the ELA (ELAGIGILTV, square/straight line), Tax (LLFGYPVYV, triangles/dashed line), LLW (LLWNGPMAV, close circles/dash‐dotted line), and LLW‐4A (LLWAGPMAV, open circles/dashed line) peptides assayed by circular dichroism spectroscopy. Lines represent data fits as described in Methods. (B) Apparent melting temperature T_m_ and van't Hoff's enthalpy of unfolding ΔH_vH_ (mean and SD). Errors bars represent S.D. resulting from the multivariable curve fitting.

To assay more directly the peptide binding affinity, we performed circular dichroism (CD) temperature melting experiments. The A2/LLW complex showed a melting temperature T_m_ of 66.5°C and transition enthalpies ΔH_vH_ of *ca*. −500 kJ/mol (Fig. 5). Thus, the A2/LLW complex is very stable when compared to other pMHC complexes that are also recognized by TRAV12‐2 positive TCRs (T_m_ / ΔH_vH_ 66.0°C/‐610 kJ/mol and 63.0°C/‐380 kJ/mol for A2/Tax from the human T‐lymphotropic virus type 1 (HTLV‐1) and A2/ELA, respectively).

### The germline‐encoded CDR1α loop of TRAV12‐2 contributes to pMHC binding

**Figure 6:**
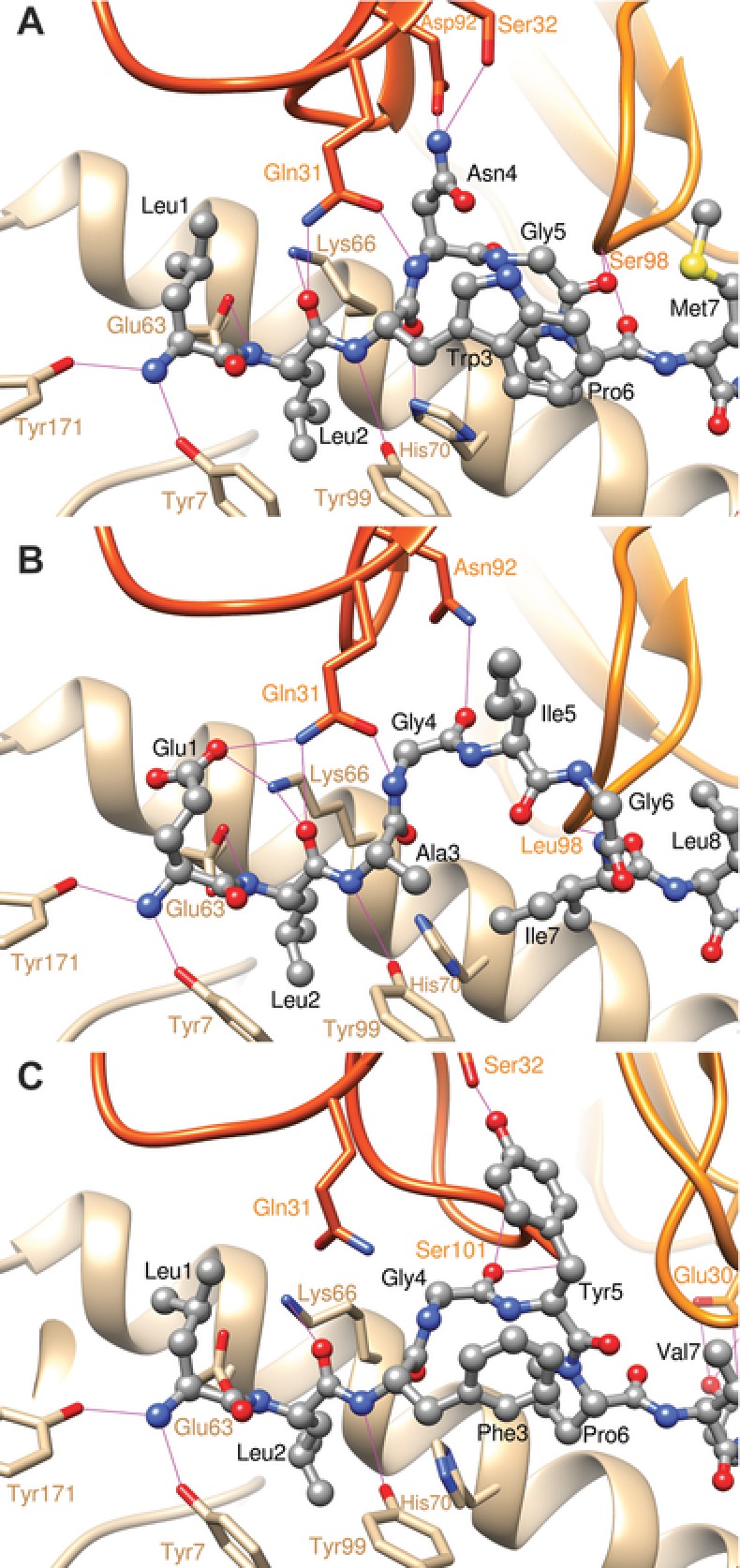
Molecular modeling indicates that germline‐encoded CDR1 in TRAV12‐2 makes major contributions to the binding with the A2/LLW complex. Calculated 3D structure of the YF5048 TRAV12/TRBV9 TCR bound to the HLA‐A2/LLW peptide complex (A) and experimental structures of the MEL5 TRAV12/TRBV30 TCR bound to HLA‐A2/ELA peptide complex, PDB ID 3G1 (B) or the HLA‐A2/Tax peptide complex, PDB ID 4FTV (C), with ribbons representing α‐ and ‐ β chains in dark and light orange, respectively; the MHC molecule in tan ribbon, and peptides in ball and stick representation. TCR and MHC side chains are shown in thick lines, with carbon atoms colored in orange and tan, respectively. Hydrogen bonds are displayed as magenta thin lines.

To investigate the structural determinants of a TRAV12‐2 positive TCR interacting with A2/LLW, we supported our experimental A2/LLW crystal data with two complementary strategies. First, we combined our structure of the A2/LLW and the previously solved structures of the MEL5 TCR to perform in silico modeling of the A2/LLW‐specific YF5048 TCR (TRAV12‐2/TRBV9; Fig. 6A and Supporting Information Table 2). This A2/LLW‐specific TCR from clone 5048 NN4 (hereafter YF5048) was chosen out of our A2/LLW‐specific clone database due to its closest similarity to the MEL5 TCR sequence for the α chain (just 3 amino acids different; Supporting Information Fig. 1A). The overall conformation of the LLW peptide binding to the MHC molecule is similar to the ELA peptide thus facilitating the modeling (Supporting Information Fig. 1B). Figure 6A and Supporting Information Table 2 show the molecular interactions taking place between the TRAV12‐2 TCR and the A2/LLW complex. In this model, most of the interactions between the TCR and the peptide originate from the α chain encoded by TRAV12‐2. Five peptide residues are pointing toward the TCR: Leu1, Asn4, Gly5, Met7 and Ala8; and these predominantly contact the CDR1α loop. In particular, Asn4 extends into a polar pocket of TRAV12, where its side chain is making a network of hydrogen bonds with the side chains of CDR1α Ser32 and CDR3α Asp92, as well as non‐polar interactions with CDR1α Gln31. In turn, the CDR3β loop of YF5048 expands between the Asn4 and Met7 peptide residues, and exchanges two hydrogen bonds between the backbone of Ser98 and the backbones of peptide residues Gly5 and Pro6 (Fig. 6A). This 3D model of A2/LLW/YF5048 presents important structural similarities with the experimental structures of the ELA and Tax peptides (Fig. 6). The backbone of the 3 first and last residues are nearly superimposed, and the corresponding side chains occupy the same MHC pockets: Leu2 of LLW, ELA and Tax in P2; Ala8 of LLW, Thr9 of ELA and Tyr8 of Tax in P8; Val9 of LLW and Tax, and Val10 of ELA in P9. In line with this, residues Leu1 of LLW, and Glu1 of ELA are facing the TCRα, while Met7 of LLW, Leu8 of ELA and Val7 of Tax are pointing toward the TCRβ. Important structural differences arise for the central residues of the peptides. While pocket P3 of MHC is occupied by Trp3 of LLW and Phe3 of Tax, it is occupied by Gly6 and part of the Ile5 backbone of the ELA peptide, due to a large backbone rearrangement. In addition, the peptide residue pointing toward TCRα and possibly making interactions is Asn4 for LLW, Ile5 for ELA and Tyr5 for Tax.

**Figure 7:**
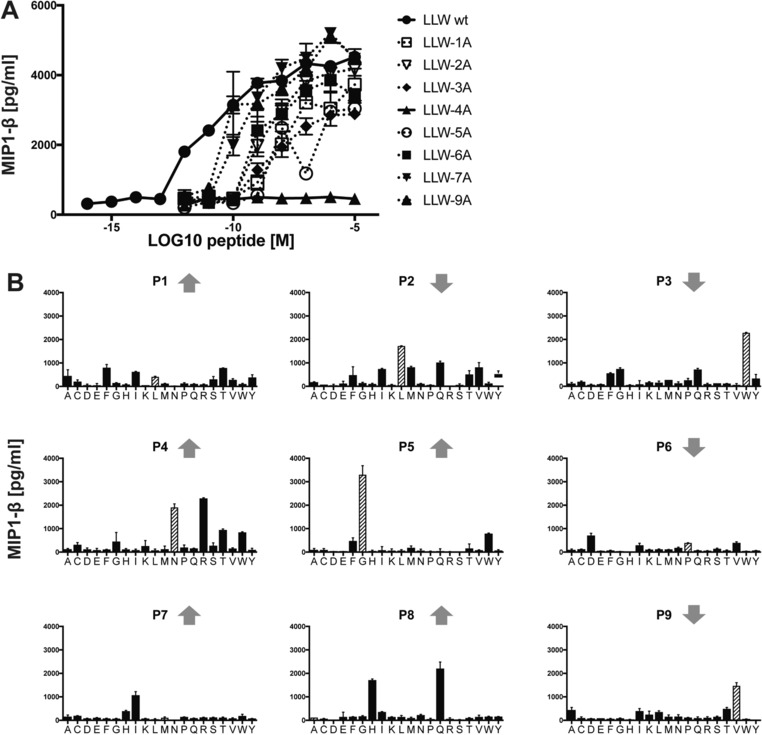
Peptide recognition signature of an individual TCR derived from clone YF5048. The A2/LLW‐specific CD8^+^ T cell clone YF5048 was functionally interrogated using amino acid substitutions of the LLW index peptide and CPL screening (A) Alanine‐scan of the LLW peptide assessed by MIP‐1β activation with graded concentrations of the peptides (mean and SEM). Data are representative of 2 independent experiments. (B) Nonamer CPL scan for clone YF5048 assayed by MIP‐1β activation (mean and SD). Index peptide residues are represented as dashed‐pattern bars. Upwards arrows (TCR‐exposed) and downwards arrows (MHC‐buried) indicate direction of amino acid side chains. Data are representative of 3 independent experiments.

The second strategy to investigate the structural determinants of the A2/LLW‐specific TRAV12‐2 was to functionally interrogate the A2/LLW‐specific CD8^+^ T cell clone YF5048 using amino acid substitutions of the LLW index peptide and combinatorial peptide library (CPL) screening. Alanine substitutions at each position of the LLW peptide revealed that the central region of the peptide (positions 3–5) was key for TCR recognition as these substitutions were deleterious to recognition; in particular the Asn4→Ala4 (LLW‐4A) was very informative as it completely abrogated the response of the YF5048 clone (Fig. 7A). This suggested that the TCR makes the majority of its critical contacts in the central region of the peptide, which is consistent with the conformation of the peptide accommodating into a central bulge (Figs. 4A and B). The dramatic effect of the Asn4→Ala4 mutation is in line with the critical interactions made by the TRAV12‐2 CDR1α loop of the TCR with the Asn (Fig. 6A and Supporting Information Table 2). Interestingly, both A2/LLW and the mutated A2/LLW‐4A complexes were found to have the same T_m_ of 66.5°C, ruling out the possibility that the absence of response to the mutated peptide LLW‐4A is a consequence of the instability of its complex with HLA‐A*02 (Fig. 5).

To further explore the observations of the alanine scan we performed a nonamer CPL screen of the YF5048 clone, which revealed positions of the peptide where index residues gave similar (positions 1, 4 and 6) or superior (positions 2, 3, 5 and 9) activation compared to non‐index amino acids (Fig. 7B). In contrast, index residues were seen minimally at positions 7 and 8, with relatively high responses seen in both cases for non‐index amino acids (Fig. 7B). In concordance with the alanine scan in the LLW peptide backbone, activation toward the index residues at positions 3 (Trp) and 5 (Gly) were dominant over the other amino acid residues. Despite the dramatic loss of YF5048 activation toward the Asn4→Ala4 in the index peptide, non‐index residues (namely Arg, Thr and Trp) also gave activation that was comparable or superior (Arg) to the Asn at position 4 (Fig. 7B). In light of this result, we performed an Asn4→Arg4 substitution of the index peptide, which ablated recognition of the peptide by YF5048 (Supporting Information Fig. 3A and 3B) and therefore supported the previous data that showed the importance of the Asn4 for TCR recognition. The loss of reactivity toward the peptide LLW**R**GPMAV suggested that the Asn4→Arg4 substitution alone was not sufficient to be seen by the TCR and required further amino acid changes at other positions of the index peptide to achieve activation. The ability of a CPL to identify amino acids that can be substituted and recognized by a clone was demonstrated using second TRAV12‐2 positive A2/LLW‐specific clone, YF5031. CPL data for YF0531 showed that this clone preferred the index residue sub‐libraries at the central positions of the peptide (Supporting Information Fig. 2A), akin to YF0548 (Fig. 7B). Maintaining this central region with substitutions at 5 other positions (**KQ**WNG**FIP**V) substitutions in bold and underlined) gave a peptide sequence that activated YF0531 (Supporting Information Fig. 3C), thereby further highlighting the importance of the central residues for TRAV12‐2 TCR recognition of the YF peptide. We also performed CPL screening of TRAV12‐2 negative clones to explore their reactivity toward the central region of the peptide. Whereas the TRAV12‐2 positive clones YF5048 (Fig. 7B) and YF5031 (Supporting Information Fig. 2A) were focused on index residues Trp3 and Gly5, TRAV12‐2 negative clone YF5001 recognized index and multiple non‐index amino acid residues at these positions. Although YF5001 recognized the Asn4 sub‐library, other amino acid sub‐libraries (Ile, Arg and Try) were of comparable or greater potency (Supporting Information Fig. 2B). The second TRAV12‐2 negative clone YF5048NN1, however, exhibited a focused recognition across the central region of the peptide; preferring only Trp (index) at position 3, and Gly (index) or Thr (non‐index) at position 5 (Supporting Information Fig. 2C). Interestingly, any response by YF5048NN1 toward the TRAV12‐2 TCR critical Asn at position 4 was unconvincing and instead dominated by activation toward the Ser‐fixed sub‐library (Supporting Information Fig. 2C). Taken together these data further support the importance of the central amino acid residues, especially the Asn4, of the LLW peptide in the binding of TRAV12‐2 positive TCRs.

In conclusion, the complementary approaches of modeling and cell functional assays demonstrated the key elements that mediate the TRAV12‐2 positive YF5048 TCR interaction with the A2/LLW complex and support that the germline‐encoded CDR1α loop of TRAV12‐2 makes critical contributions to cognate peptide recognition.

### A2/LLW and A2/ELA TRAV12‐2 positive TCRs preserve their respective specificity

**Figure 8:**
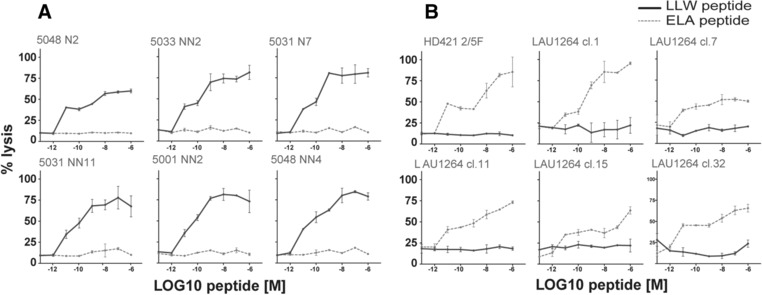
Absence of cross‐reactivity between A2/LLW and A2/ELA epitopes. Recognition of the LLW and ELA peptides by A2/LLW‐specific and A2/ELA‐specific CD8^+^ T cell clones was assessed by chromium release assay. (A) Cross‐reactivity analysis of TRAV12‐2 positive A2/LLW‐specific CD8^+^ T cell clones toward the LLWNGPMAV (LLW, solid line) and ELAGIGLTV (ELA, dashed line) (mean and SD). (B) Cross‐reactivity analysis of TRAV12‐2 positive A2/ELA‐specific CD8^+^ T cell clones toward the LLWNGPMAV (LLW, solid line) and ELAGIGLTV (ELA, dashed line) (mean and SD).

**Figure 9:**
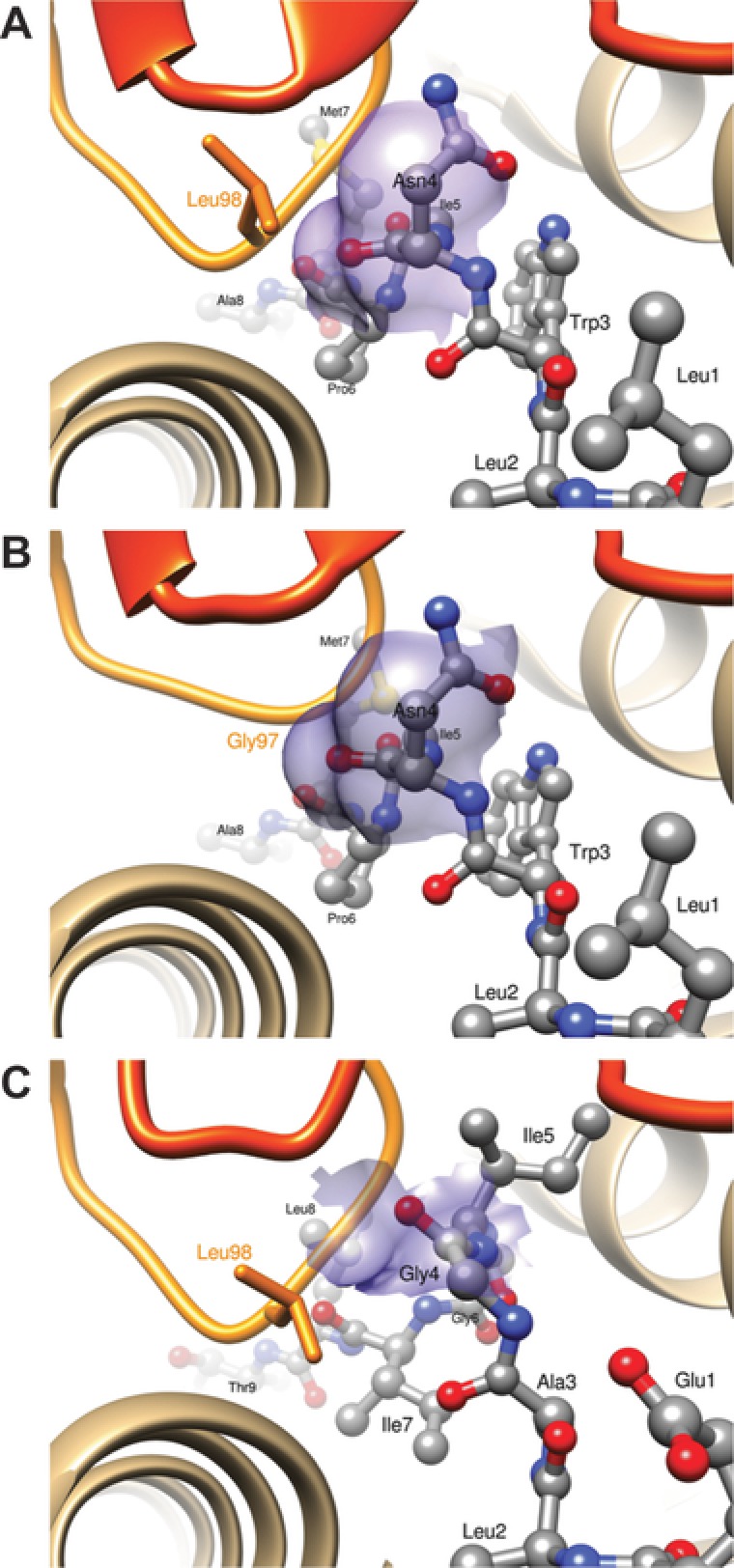
Calculated structures of the MEL5 and YF50408 TCRs bound to the HLA‐A2/LLW peptide complex and the experimental 3D structure of the MEL5 TCR bound to the HLA‐A2/ELA peptide complex. Calculated structures of the MEL5 and YF50408 TCRs bound to the HLA‐A2/LLW peptide complex, (A) and (B), respectively. Experimental 3D structure of the MEL5 TCR bound to the HLA‐A2/ELA peptide complex (C). Representation: α and β chains are in dark and light orange ribbons, respectively; the MHC molecule in tan ribbon; peptides in ball and stick; TCR side chains in thick lines representation, with carbon atoms colored in orange; the transparent surfaces of Asn4 (of LLW) or Gly4 (of ELA), in contact with CDR3 Leu98 (MEL5) or Gly97 (YF5048), are colored in magenta.

Given the germline nature of the CDR1α loop of TRAV12‐2 that is critical to peptide recognition of both A2/LLW and A2/ELA specificities, we addressed whether there was any cross‐reactivity between T‐cells with these TRAV12‐2‐dominated specificities. The TRAV12‐2 positive A2/LLW‐specific clones did not respond to the ELA peptide (Fig. 8A) and the TRAV12‐2 positive A2/ELA‐specific clones did not respond to the LLW peptide (Fig. 8B) indicating there was no common, shared TRAV12‐2‐mediated mode of antigen recognition. These functional data were supported by in silico modeling of the MEL5 TCR together with the A2/LLW complex showing unfavorable interactions (Fig. 9A). In fact, the most important difference in the interaction scheme of A2/LLW with the A2/LLW‐specific YF5048 TCR versus with the A2/ELA‐specific MEL5 TCR involves the CDR3β loop. In the A2/LLW/MEL5 structural model, the Asn4 residue of the LLW peptide is situated close to the Leu98 residue of the CDR3β loop. The contact between the backbone carbonyl of Asn4 and the side chain of Leu98 prevent the former from making any hydrogen bond with its surrounding, and is unfavorable to the binding between A2/LLW and MEL5 (Fig. 9A). In contrast, in the A2/LLW/YF5048 structural model, the key residue in CDR3β loop is Gly97, which does not sterically hinder the backbone carbonyl of the peptide Asn4 residue (Fig. 9B). In the A2/ELA/MEL5 structure, due to a different position of the peptide backbone, the Gly4 residue of ELA (corresponding to Asn4 in LLW) is situated far from Leu98 in the CDR3β loop, and its backbone carbonyl is unhindered by this non‐polar side chain (Fig. 9C). This key difference in the CDR3β loops explains the unfavorable interaction between MEL5 and A2/LLW, compatible with the lack of cross‐reactivity observed between TRAV12‐2 positive TCRs toward LLW or ELA. These data highlight the importance that the CDR3β loop plays in TCR specificity as although this loop plays a minimal role in pMHC contact it can act to interfere with engagement.

## Discussion

In this study, we analyzed the TCR repertoire of CD8^+^ T cells specific for the immunodominant A2/LLW epitope in YF‐17D vaccinees and controls. We revealed and quantified the TRAV12‐2 bias in A2/LLW‐specific CD8^+^ T cells. Various functional assays using T‐cell clones demonstrated that TRAV12‐2 does not provide a functional advantage on a per cell basis. Together with the fact that this strong TRAV12‐2 bias was already present in naïve A2/LLW‐specific CD8^+^ T cells before YF‐17D vaccination, it rather suggests that TRAV12‐2 might confer a selective advantage for high frequency and prevalence by favoring thymic output of naïve cells. We thus sought to investigate how TRAV12‐2 may provide such advantage by investigating the mode of antigen binding and structural considerations of the TCR‐peptide‐MHC complex.

The A2/ELA epitope represents a well‐known model antigen for which T cells are biased for TRAV12‐2 usage 13, 14, 15, 16, 17. A2/ELA‐specific CD8^+^ T cells exhibit high frequency and prevalence in HLA‐A*0201 healthy individuals as well as melanoma patients, showing naïve and differentiated phenotypes, respectively. Intriguingly, the binding between the MEL5 TCR expressing TRAV12‐2 and the ELA peptide in complex with HLA‐A*0201 occurs via dominant contacts with the CDR1 loop of TRAV12‐2 13, 18, 21, 22. The TRAV12‐2 gene is also expressed by the A6 TCR, which is specific for the A2/Tax epitope of the HTLV‐1 4. The CDR1α and CDR2α loops of the A6 TCR utilize an antigen‐binding mode virtually identical to that seen in the MEL5‐A2/ELA complex, making contacts between the CDR1α loop and the Tax peptide. A study in HTLV‐I‐Associated Myelopathy/Tropical Spastic Paraparesis (HAM/TSP) patients revealed that TRAV12‐2 transcripts are predominant 23 and the frequency of naïve cells with this specificity is very high 24. Therefore, A2/Tax‐specific CD8^+^ T cells constitute another documented example of high naïve frequency associated with TRAV12‐2 bias.

Unfortunately, our extensive attempts to generate a TRAV12‐2 TCR A2/LLW co‐crystal structure failed so we resorted to molecular modeling of this interaction taking advantage of the high sequence similarity between the A2/LLW‐specific TCR YF5048 and the A2/ELA‐specific TCR MEL5. Conveniently, the LLW peptide in the free A2/LLW structure we solved adopts a similar conformation to the ELA peptide in the A2/ELA/MEL5 TCR complex. Modeling showed that the YF5048 TCR α‐chain positioned above the N‐terminus of the peptide, making contacts predominantly with Asn4 in the middle of the peptide via the CDR1α loop of TRAV12‐2. The importance of this interaction is further supported by our results from the mutagenesis scan across the LLW peptide and CPL screen, highlighting Asn4 as a critical residue for TCR recognition by TRAV12‐2 positive TCRs. Our modeling data suggests that the germline‐encoded TRAV12‐2 CDR1α loop of A2/LLW‐specific CD8^+^ T cells makes critical contacts with both MHC and peptide in a comparable manner to the CDR1α loops in the MEL5 and A6 TRAV12‐2 positive TCRs; the MEL5 and A6 4, 18. These three paralleled examples of TRAV12‐2 biased responses endorse the concept that the interactions between the TCR and the antigen can rely substantially on TCR segments that already pre‐exist in the germline rather than on somatic CDR3 rearrangement. However, it is important to note that this observation does not apply to all immunodominant T cell responses, as many public TCRs or immunodominant epitope‐specific TCRs bind their cognate peptide predominantly via residues encoded in the CDR3 rearranged loops 6, 25.

Importantly, we showed that TCRs sharing this heritable TRAV12‐2 CDR1α component of antigen binding still preserve their respective antigen specificity. Indeed, we demonstrated that there is no cross‐reactivity between the LLW and ELA specificities. Based on in silico modeling, the CDR3β loop sterically hindered engagement of the non‐cognate peptide. Thus, even these examples of a TRAV germline‐encoded antigen binding mode are still heavily reliant on permissive sequences within the TRBV non‐germline CDR3 loop.

It is intriguing that these three examples of TCRs binding their epitope with a germline component all involve the CDR1α loop of TRAV12‐2 and HLA*0201. It is conceivable that TCRs expressing the TRAV12‐2 could have a selective advantage for binding to cognate antigen restricted by HLA‐A*0201 or that other antigen specificities (not only restricted by HLA‐A*0201) also harbor biases for certain germline‐encoded TCR segments but that these have not yet been identified. The HLA‐A*0201 allele and its associated antigen specificities are the most studied because HLA‐A*0201 is prevalent at 30–50% in Caucasian populations and is the most prevalent HLA subtype amongst the global human population, potentially inducing a research bias 26. Indeed, the TCR/pMHC structural database is dominated by interactions with HLA A2. More studies need to be conducted to appreciate the extent to which this phenomenon of germline‐encoded TCR recognition applies to other specificities and TRAV/TRBV families.

Despite the tremendous theoretical genetic diversity of the TCR repertoire, most studies showed that the adult TCR repertoire is a consequence of a process that is far from random and TCR bias is commonly found in immune responses 27. A specificity and/or TCR bias could reflect an evolutionary advantage during infection and other diseases. Several lines of evidence indicate that the germline‐encoded TCR segments have features that promote binding to MHC molecules, suggesting co‐evolution between TCR and MHC molecules 28, 29, 30. Our data suggests that there is also co‐evolution between the TCR and the cognate peptide. Indeed, we observed that TRAV12‐2 TCR bias is present before YF‐17D vaccination. In agreement with our functional studies on A2/LLW‐specific clones, it was reported that TRAV12‐2 usage in A2/ELA‐specific CD8^+^ T cells was independent from functional avidity 15. In fact, the origin of the large naïve A2/ELA‐specific CD8^+^ T cell population was attributed to preferential thymic selection 31, 32. Given that antigen recognition features a germline‐encoded component, there is presumably a genetic advantage that confers higher chances for thymic output of TCR constructions involving the CDR1α of TRAV12‐2. Thus, although TRAV12‐2 does not confer a functional advantage on a per cell basis, it may provide an advantage at the level of the organism by skewing the naïve CD8^+^ T cell compartment toward these specificities recognized by TRAV12‐2 CDR1α. This possibly explains the high frequency and prevalence of specificities such as A2/LLW and A2/ELA.

In summary, we discovered the TCR bias for TRAV12‐2 in A2/LLW‐specific CD8^+^ T cells and demonstrated that there is no functional advantage in featuring TRAV12‐2 on a per cell basis. Rather, our structural modeling suggests that the germline‐encoded CDR1α loop centrally contributes to peptide binding similar to two other TRAV12‐2 positive TCR specificities. We also demonstrated that TCRs sharing this TRAV12‐2 CDR1α – mediated mode of antigen binding still preserve their own antigen specificity.

## Materials and methods

### Peripheral blood samples

All PBMC samples were obtained in the framework of our previously published clinical study, approved by the Human Research Ethics Committee of the Canton de Vaud (protocol 329/12) with healthy volunteers participating under written informed consent 8.

### Generation of T cell clones

All A2/LLW‐specific CD8^+^ T cell clones used in this study were generated in the laboratory of D. Speiser, derived from 4 healthy YF‐17D (Stamaril, Sanofi Pasteur) vaccinees. Purified A2/LLW tetramer‐positive populations were isolated by FACS as described 8 and cloned by limiting dilution in Terasaki plates, cultured in RPMI 1640 medium supplemented with 8% human serum and 150 U/mL recombinant human IL‐2 (rIL‐2). Thereafter, T cell clones were expanded by periodic restimulation with 1 μg/mL PHA and 10^6^/mL irradiated allogeneic PBMC (30 Gy) as feeder cells. The MEL5 clone was generated in the laboratory of A. Sewell as previously described 18. The clones HD421 2/5F and LAU1264 were generated in the laboratories of D. Speiser and N. Rufer as previously described 15.

### TCR repertoire and clonotype analysis in A2/LLW‐specific CD8^+^ T cell clones

Total RNA was isolated using the PicoPure RNA kit per manufacturer's instructions, and cDNA prepared and sequenced as previously described 21. Briefly, for the Vβ repertoire, each cDNA sample was subjected to individual PCRs using a set of previously validated forward primers specific for the 22 TRBV subfamilies and one reverse primer specific for the corresponding Cβ gene segment. For the Vα repertoire, we amplified and sequenced the TRAV12‐2 segment using the TRAV12 (forward) and TRAC (reverse) primers. PCR amplicons of interest were sequenced from the reverse primer by Fasteris S.A. TRAV and TRBV segments were described according to the IMGT nomenclature 33.

### ^51^Chromium release assays

The HLA‐A*02^+^ human mutant cell line CEMx721.T2 (American Type Culture Collection) was used as target by labeling with ^51^Cr for 1 h at 37°C, followed by extensive washing. Target cell killing was assessed by chromium release in the supernatant upon co‐culture with CD8^+^ T cell clones (effector cells) at the Effector:Target ratio of 10:1 for 4 h at 37°C in V‐bottom microwells, in presence of serial dilutions of the peptide (LLWNGPMAV or ELAGIGILTV), measured using a gamma counter and calculated as:

$$\%\;\mathrm{specific}\;\mathrm{lysis}=100\times \frac{\left(\text{e}xperimental-\text{s}pontaneous\;release\right)}{\left(\text{t}otal-\text{s}pontaneous\;release\right)}$$

### Flow cytometry

CD8^+^ T cells were first enriched from cryopreserved samples using the human CD8^+^ T cell enrichment kit from StemCell Technologies (negative selection, per manufacturer's instructions). Stainings were performed using phosphate‐buffered saline with 5 mM EDTA, 0.2% bovine serum albumin, and 20 mM sodium azide [fluorescence‐activated cell sorting (FACS) buffer]. Tetramer stainings were performed for 40 min at 4°C. All tetramers were purchased from TCmetrix Sàrl : HLA‐A*0201/LLWNGPMAV (NS4b^214‐222^, Yellow Fever Virus), HLA‐A*0201/VMLFILAGL (NS4a^54‐62^, Yellow Fever Virus), HLA‐B*0701/RPIDDRFGL (NS5^211‐219^, Yellow Fever Virus), HLA‐A*0201/GLCTLVAML (BMFL12^80‐288^, Epstein Barr Virus), HLA‐A*0201/NLVPMVATV (pp65^495‐503^, Cytomegalovirus), HLA‐A*0201/ELAGIGILTV (Melan‐A^26‐35 (A27L)^, Melanoma). Surface antibody staining was then performed, followed by staining with LIVE/DEAD‐Fixable‐Aqua (Invitrogen), each step at 4°C for 30 min. Cells were fixed overnight in 0.36% formaldehyde (supplemented with 2% glucose and 5 mM sodium azide). Samples were acquired using a Gallios flow cytometer (Beckman Coulter, three‐laser configuration). The data were processed with FlowJo (Tree Star Inc., v9.5.2). Samples with antigen‐specific populations below 0.001% tetramer‐positive cells in total CD8^+^ T cells were considered negative and populations consisting of less than 20 events were not considered eligible for further analysis.

### Intracellular cytokine staining assay

A2/LLW‐specific CD8^+^ T cell clones were stimulated with LLW peptide‐loaded T2 cells at the E:T cell ratio of 1:2 for 4 h at 37°C in the presence of Brefeldin‐A (Sigma‐Aldrich) and anti‐CD107a‐FITC antibody (BD Biosciences). Then, cells were stained with anti–CD8–APC‐AF750 antibody (Beckman Coulter) at 4°C for 30 min. After washing in PBS, cells were incubated with LIVE/DEAD‐Fixable‐Aqua (Invitrogen) at 4°C for 30 min, and fixed at 4°C overnight (0.36% formaldehyde buffer). Cells were washed and stained intracellularly with anti‐IFNγ‐PC7, anti‐TNFα‐A700 and anti‐IL‐2‐PerPCP‐Cy5.5 antibodies (BD Biosciences) in FACS buffer with 0.1% saponin for 30 min at 4°C. Samples were acquired and data processed as described above.

### NTAmer staining and dissociation kinetic measurements

Dually labeled pMHC multimers built on NTA‐Ni^2+^‐His‐tag interactions called NTAmers (synthetized by TCMetrix Sàrl) were used for dissociation kinetic measurements 34, 35. Stainings with dually PE‐ and Cy5‐labeled A2/LLW‐specific NTAmers and data analysis were done as previously described 34, 36. Briefly, staining was measured at 4°C using a thermostat device on a SORP‐LSR II flow cytometer (BD Biosciences). Following 30 s of baseline acquisition, imidazole (100 mM) was added. PE and Cy5 fluorescence were measured during the following 5 min. Data were processed using the kinetic module of the FlowJo software (v.9.7.6; Tree Star), and corrected mean fluorescence intensity values were plotted and analyzed using the GraphPad Prism software (v.6; GraphPad).

### Combinatorial peptide library (CPL) scans

The nonamer CPL contains a total of 4.8 × 10^11^ ((9+19) × 19^8^) different nonamer peptides divided into 180 sub‐libraries with each containing 19^8^ different nonamer peptides in approximately equimolar concentrations (Pepscan, Lelystad, The Netherlands) 37. Each of the 180 sub‐libraries has a fixed amino acid residue but all other positions are degenerate. Cysteine was excluded from all degenerate positions to avoid oxidation, but was included at the fixed positions. Prior to the assay, CD8^+^ T cell clones were washed and rested overnight in R5 medium (RPMI 1640 supplemented with 100 units/mL penicillin, 100 μg/mL streptomycin, 2 mm l‐glutamine, and 5% heat‐inactivated fetal calf serum (all Invitrogen). For CPL screening, 6 × 10^4^ C1R A2 cells 38 were pulsed with each sub‐library at 100 μg/mL in duplicate for 2 h at 37°C. After peptide pulsing, 3 × 10^4^ CD8^+^ T cells were added, and the cultures were incubated overnight at 37°C. Subsequently, the supernatant was harvested and assayed for MIP‐1β by ELISA according to the manufacturer's instructions (R&D Systems).

### Protein expression, refolding, and purification

HLA A*0201 α‐chain and β2m were expressed separately, without post‐translational modification, as insoluble inclusion bodies (IBs) in competent Rosetta (DE3) *Escherichia coli* cells, using 0.5 M IPTG to induce expression as thoroughly described recently 39. Briefly, for a 1L pMHC refold, 30 mg HLA‐A*0201 α‐chain was mixed with 30 mg β2 m and 4 mg peptide at 37°C for 15 min with 10 mM DTT. This mixture was then added to cold refold buffer (50 mM Tris, pH8, 2 mM EDTA, 400 mM L‐arginine, 6 mM cysteamine hydrochloride, and 4 mM cystamine). Refolds were mixed at 4°C for > 6 h. Dialysis was performed against 10 mM TRIS, pH8.1, until the conductivity of the refolds was less than two millisiemens per centimeter. The refolds were then filtered and purified first by ion exchange using a Poros50HQTM column (GE Healthcare, Buckinghamshire, U.K.) and second by gel filtration directly into crystallization buffer (10 mM Tris pH8.1 and 10 mM NaCl) or PBS buffer (137 mM NaCl, 3 mM KCl, 8 mM Na_2_HPO_4_, 1 mM KH_2_PO_4_) using a Superdex200HRTM column (GE Healthcare, Buckinghamshire, U.K.). Protein quality was analyzed by Coomassie‐stained SDS‐PAGE, either under non‐reducing or reducing conditions. HLA‐A*0201 was refolded with the peptides LLWNGPMAV (A2/LLW) or LLWAGPMAV (A2/LLW‐4A).

### Crystallization, diffraction data collection, and model refinement

All protein crystals were grown at 18°C by vapor diffusion via the “sitting drop” technique. 200 nL of each pMHC (20 mg/mL) in crystallization buffer was added to 200 nL of reservoir solution. A2/LLW crystals were grown in 0.1 M Hepes, pH7, 0.2 M ammonium sulphate, 20%PEG 4000. All crystals were soaked in 30% ethylene glycol before cryo‐cooling. All crystallization screens and optimization experiments were completed using an Art‐Robbins Gryphon dispensing robot (Alpha Biotech Ltd., UK). Data were collected at 100 K at the Diamond Light Source (Oxfordshire, UK) as described previously 40. All data sets were collected at a wavelength of 0.98 Å using an ADSC Q315 CCD detector. Reflection intensities were estimated with the XIA2 package, and the data were scaled, reduced, and analyzed with the SCALA and CCP4 package. Structures were solved with molecular replacement using PHASER. A solution could be obtained with a search model taken from Protein Data Bank entry 5EU5. Sequences were adjusted with COOT, and the models were refined with REFMAC5. Graphical representations were prepared with PyMOL. The reflection data and final model coordinates were deposited in the Protein Data Bank (PDB code: 5N6B).

### Measuring the thermal stability of HLA‐A*0201–peptide complexes

Thermal stability of A2/peptide complexes was assessed by CD spectroscopy monitoring the change of ellipticities Θ at 218nm where the spectra exhibit a minimum. Data were collected on an Aviv 215 spectrometer (Aviv Biomedical Inc., Lakewood, NJ) equipped with a thermostated cell holder using a 1 mm quartz cell. Proteins were dissolved in PBS at c = 3.5 μM. Denaturation was monitored from 4°C up to a temperature when protein precipitation occurred using a gradient of 0.5°C/min. Melting curves were analyzed assuming a two‐state native (N) to denatured (D) transition N_3_ ↔ 3D with the melting temperature and van't Hoff's enthalpy at the midpoint of the transition as fitting parameters 41, 42.

### Modeling the TCR‐p‐MHC complex

The 3D structure of the TRAV12‐2/TRBV9 TCR in complex with HLA‐A2 and the LLWNGPMAV peptide was modeled from three experimental structures: 3HG1 18 and 4QOK 43, containing a complex between the TRAV12‐2/TRBC1 TCR in complex with HLA‐A2 and the ELAGIGILTV or EAAGIGILTV peptides, respectively, and the experimental structure obtained in this study for the complex between HLA‐A2 and the LLWNGPMAV peptide. The sequence alignment between TRBV9 and TRBC1 was performed using the MUSCLE program 44. The sequence identity between the variable part of the TRBV9 and TRBC1 TCR beta chains is 30%. Based on this sequence alignment, the model was obtained using the Modeller program 45, 46. 1000 models were generated by satisfaction of spatial restraints through minimization and simulated annealing, and the model with the best Modeller objective function was retained. Molecules were visualized and analyzed using UCSF Chimera 47.

The model of A2/LLW in complex with the MEL5 TCR was obtained with the Modeller program, following the method described above to obtain the structural model of A2/LLW with the YF5048 TCR. In this case, however, we used our experimental structure of the A2/LLW as a template for the HLA/peptide domain and the experimental structure of the complex between A2/ELA and MEL5 (PDB code: 3HG1) as a template for the MEL5 TCR.

## Conflict of interest

The authors declare no commercial or financial conflict of interest.

## Supporting information

Peer review correspondence

Supporting Information
