# Supplementary material for: T cell receptor alpha variable 12‐2 bias in the immunodominant response to Yellow fever virus
Source: Eur J Immunol. 2017 Dec 11;48(2):258–72. doi: 10.1002/eji.201747082 (PMC5887915; doi:10.1002/eji.201747082)
Supplement: Supplementary file 2 — Supporting Information [file EJI-48-258-s002.pdf]

European Journal of Immunology

Supporting Information
for
DOI 10.1002/eji.201747082

Amandine Bovay, Vincent Zoete, Garry Dolton, Anna M. Bulek, David K. Cole, Pierre J. Rizkallah, Anna Fuller, Konrad Beck, Olivier Michielin, Daniel E. Speiser, Andrew K. Sewell and Silvia A. Fuertes Marraco

T cell receptor alpha variable 12-2 bias in the immunodominant response to Yellow fever virus

© 2017 WILEY-VCH Verlag GmbH & Co. KGaA, Weinheim

Supporting Information for:

T cell Receptor Alpha Variable 12-2 bias in the immunodominant response to Yellow fever Virus

Amandine Bovay, Vincent Zoete, Garry Dolton, Anna M. Bulek, David K. Cole, Pierre J. Rizkallah, Anna Fuller, Konrad Beck, Olivier Michielin, Daniel E. Speiser, Andrew K. Sewell, Silvia A. Fuertes Marraco#

# Corresponding author: silviaasuncion.fuertesmarraco@unil.ch

This PDF file includes:

Table S1. Analysis of the relative frequencies of T cell clonotypes within A2/LLW-specific CD8 T cells.

Table S2. Molecular interactions between TCR and pMHC in the structural model.

Fig S1. High similarity between the YF5048 and MEL5 TCRs and their cognate pMHC complexes.

Fig S2. Peptide recognition signature of individual TCRs derived from TRAV12-2 positive and negative clones specific for A2/LLW.

Fig S3. Effect of the single substitution Asn4 -> Arg4 on clone YF5048.

Table S1. Analysis of the relative frequencies of T cell clonotypes within A2/LLW-specific CD8 T cells.[1]

TRAV	CDR3	TRAJ	Frequency
12-2	CAVTDDKIIFG	30	0.11
12-2	CAVDSGGYQKVTFG	13	single
12-2	CAGGDDKIIFG	30	single
12-2	CAVKDARLMFG	31	0.05
12-2	CAVGSDKIIFG	30	0.07
12-2	CAVSDARLMFG	31	single
12-2	CAAFDDKIIFG	30	0.05
12-2	CAASASKLIFG	4	single
12-2	CAVDTNAGKSTFG	27	single
12-2	CAAVNNDMRFG	43	single
12-2	CAVGDARLMFG	43	single
12-2	CAANNARLMFG	31	single
12-2	CAVNGNKLVFG	47	single
12-2	CAVGDDKIIFG	30	0.09
12-2	CAVNSNTDKLIFG	34	single
12-2	CAVTTSGTYKYIFG	40	single
12-2	CAATDDKIIFG	30	single
12-2	CAVNDKYKLSFG	20	single
12-2	CAGSGDKLIFG	34	single
12-2	CAEEGRGNDMRFG	43	single
12-2	CAVSKDDKIIFG	30	single
12-2	CASRDDKIIFG	30	single
12-2	CAVTPDKVIFG	50	single
12-2	CAVTSDSWGKLQFG	24	0.04
12-2	CAVVDDKLIFG	4	single
12-2	CAVRDDKIIFG	30	single
12-2	CATSNNRLAFG	7	single
12-1	CVVNADDMRFG	43	single
12-1	CGVDDKIIFG	30	single
12-1	CVVTGTYKYIFG	40	0.05
12-1	CVVGTDKLIFG	34	single
13-1	CAASGTGAGSYQLTF	28	single
27	CAASSLYGQNFVF	26	single
19	CALSENNDYKLSF	20	0.05
22	CAVVPNAGGTSYGKLTF	52	single

TRBV	CDR3	TRBJ	Frequency
6-8	CASSYSRTGSYEQYFG	2-7	single
7-2	CASSQGLAYEQFFG	2-1	single
9	CASSVEGPGELFFG	2-2	single
2	CASSEATGASYEQYFG	2-7	0.07
2	CASSEYVQYYGYTFG	1-2	single
6-8	CASSEAGQAYEQYFG	2-7	single
9	CASSEGGQAYNEQFFG	2-1	0.07
9	CASSVSGSSYEQYFG	2-7	single
9	CASSVGTSSYEQYFG	2-7	single
9	CASSVAGGYEQYFG	2-7	single
4-3	CASSPGLAGGYEQFFG	2-1	0.05
28	CASSPPGTGVYGYTFG	1-2	0.05
6-8	CASSYSGGAYGYTFG	1-2	single
27	CASSQDPGGPSRTQYFG	2-5	single
6-8	CASSYSGGSYIEFFG	2-1	single
2	CAVGDRGYEQYFG	2-7	single
9	CASSTGLAYEQFFG	2-1	single
9	CASSVGGVVYNEQFFG	2-1	single
2	CASDSGDHEQYFG	2-7	0.05
19	CASSIGPGLGQPQHFG	1-5	single
27	CASSLNPSTDTQYFG	2-7	single
19	CASSIWRRGWLHLRFG	1-6	single
2	CASSEYVQYYFYTFG	1-2	single
9	CASSAGTGAYEQYFG	2-7	single
9	CASSATSGGADTQYFG	2-7	single
18	CASGPGTVSYEQYFG	2-7	single
29-1	CSVPFAGADTQYFG	2-7	single
28	CASSSLGTGGYGYTFG	1-2	single
2	CASNQGGISYGYTFG	1-2	single
9	CASSVATEGYGYTFG	1-2	0.07
6-8	CASSSRVGNIAGELFFG	2-2	single
9	CASSPSGGGYEQYFG	2-7	single

[1] TRAV, TRAJ, TRBV, TRBJ segment usage and relative frequencies are indicated for all clonotypes. Public sequences are colored in yellow.

Table S2. Molecular interactions between TCR and pMHC in the structural model.[2]

CDR loop	TCR residue	Peptide residue	MHC residue	Bond type
CDR1α	Arg28, Nϵ		Glu166, Oϵ1/Oϵ2	Electrostatic
	Arg28, NH2		Glu166, Oϵ1/Oϵ2	Electrostatic
	Arg28, O		Trp167, Nϵ1	Electrostatic
	Gly29, Cα		Trp167, CZ2	vdW
	Gly29, Cα	Leu1, Cδ2		vdW
	Gln31, Nϵ2	Leu2, O		Electrostatic
	Gln31, Cβ	Asn4, Cβ/Cγ		vdW
	Gln31, Cγ	Asn4, Cγ		vdW
	Gln31, Oϵ1	Asn4, N		Electrostatic
	Ser32, Oγ	Asn4, Nδ2		Electrostatic
CDR2α	Tyr51, OH		His151, O	Electrostatic
CDR3α	Asn92, Oδ2	Asn4, Nδ2		Electrostatic
	Asn94, O		Arg65, NH2	Electrostatic
	Ala95, O		Arg65, Nϵ	Electrostatic
	Ala95, Cβ		Gly62, Cα/O	vdW
	Ala95, Cβ		Lys66, Cϵ	vdW
CDR2β	Asn48, Oϵ1		Arg65, NH1	Electrostatic
	Tyr49, Cϵ1		Arg65, Cδ	vdW
	Arg55, NH2		Glu19, Oϵ1	Electrostatic
CDR3β	Gly97, Cα		Thr73, Cγ2	vdW
	Ser98, N	Gly5, O		Electrostatic
	Ser98, N	Pro6, O		Electrostatic
	Ser98, Cβ	Met7, Cϵ		vdW

[2] vdW: van der Waals

A

TCR alpha V domain	CDR1 CDR2 CDR3
MEL5 TRAV12-2	KEVEQNSGPL SVPEGAIASLNCTYS**DRGSQS**FFWYRQYSG KSPELIMS**IYSNGD**KEDGRFTAQLNKASQYVSLLIRDSQPSDSATYL**CAVNVAGKST**FG
YF5048 TRAV12-2	KEVEQNSGPLSVPEGAIASLNCTYS**DRGSQS**FFWYRQYSG KSPELIMS**IYSNGD**KEDGRFTAQLNKASQYVSLLIRDSQPSDSATYL**CAVNVAGKST**FG

B

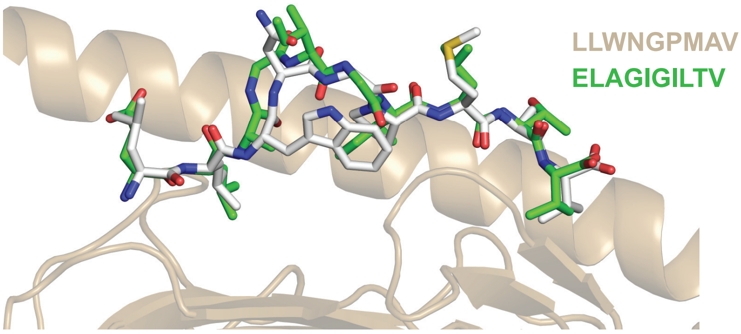

Fig S1. High similarity between the YF5048 and MEL5 TCRs and their cognate pMHC complexes.

(A) Alignment of the variable region of the TCR alpha chain of YF5048 and MEL5. (B) Superimposition of the peptides LLWNGPMAV in grey and ELAGIGILTV in green binding to the HLA-A2 molecule.

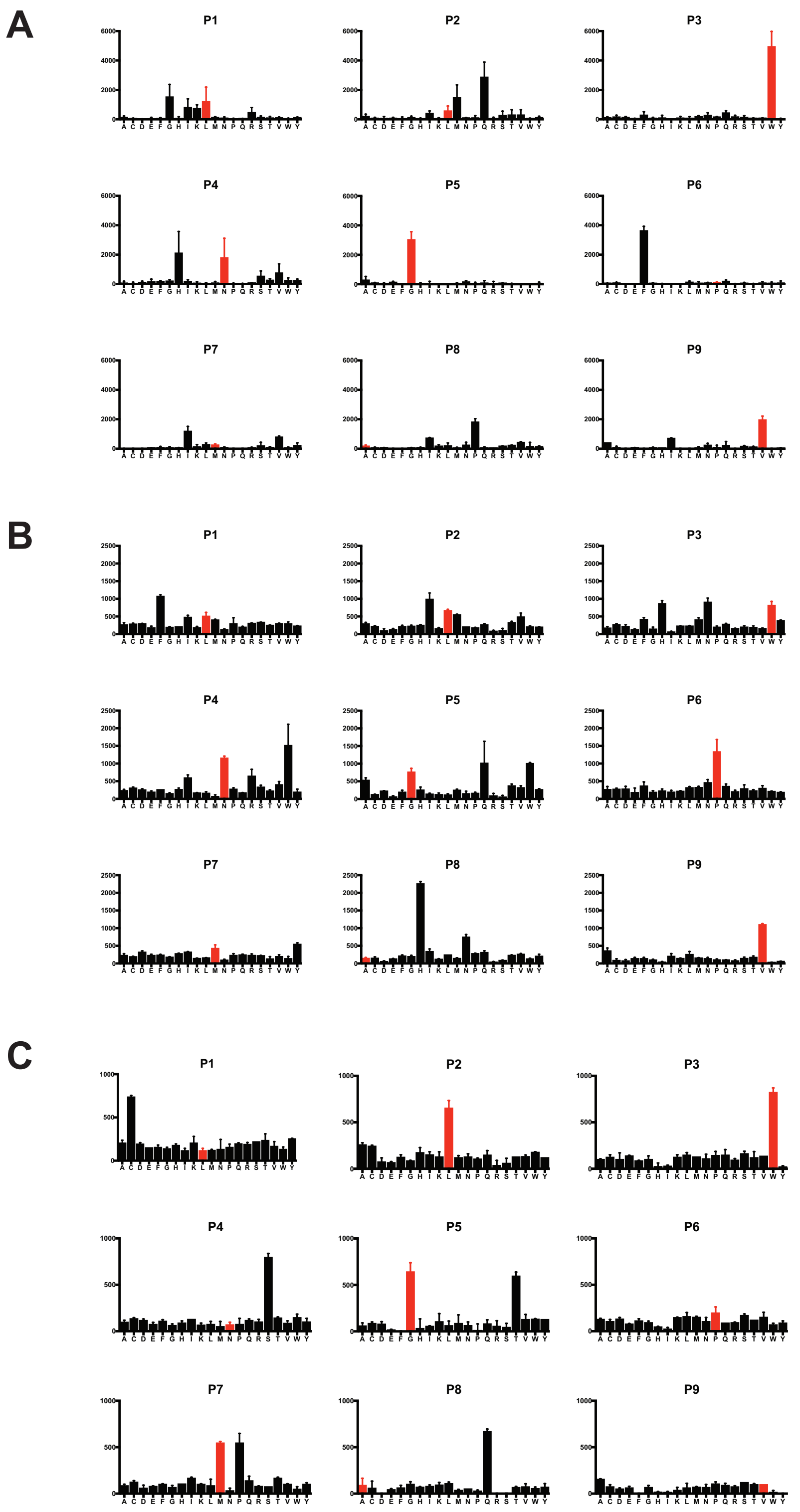

Fig S2. Peptide recognition signature of individual TCRs derived from TRAV12-2 positive and negative clones specific for A2/LLW.

Nonamer CPL scan for a TRAV12-2 positive A2/LLW-specific clone YF5031 (A), and for two TRAV12-2 negative A2/LLW-specific clones YF5001 (B) and YF5048NN1 (C) assayed by MIP-1β activation (mean and SD).

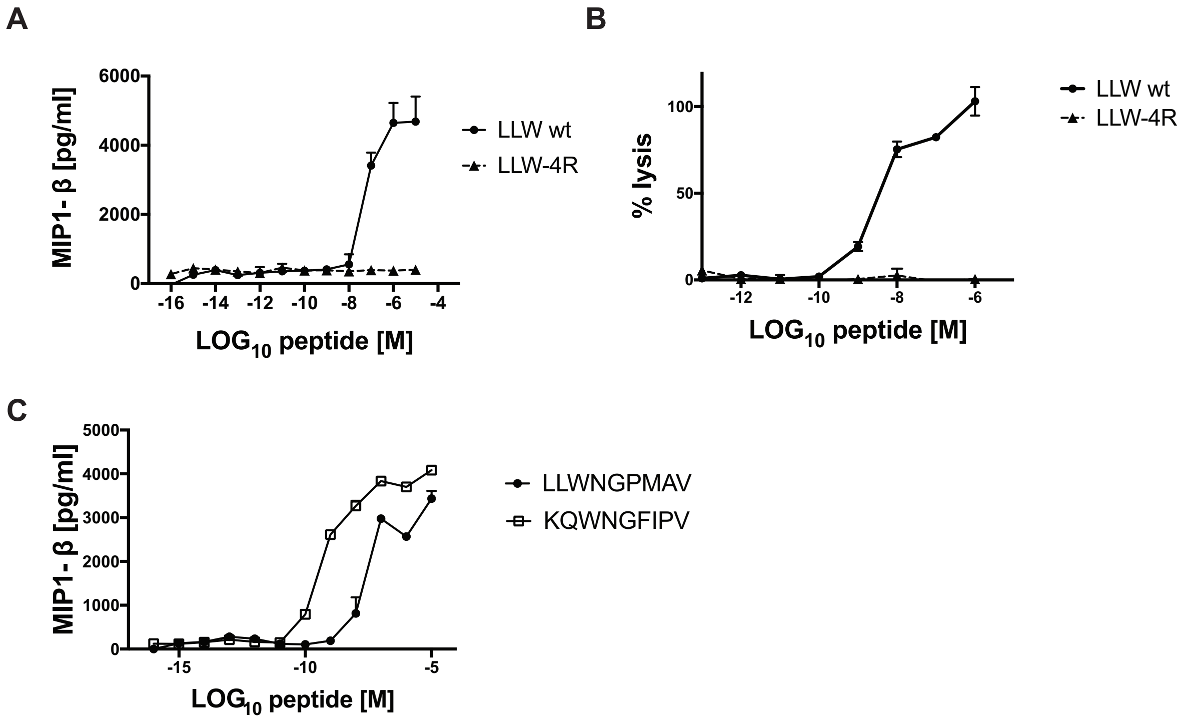

Fig S3. Effect of the single substitution Asn4 -> Arg4 on clone YF5048.

(A) Effect of the Asn4 -> Arg4 mutation on clone YF5048 assayed by MIP-1β activation with graded concentrations of the peptides LLWNGPMAV (LLW wt) or LLWRGPMAV (LLW-4R) (mean and SEM). (B) Killing capacity (51-chromium release assay) of clone YF5048 with titration of the LLW-wt or LLW-4R peptides (mean and SEM). (C) Effect of a strong agonist peptide on the TRAV12-2 positive clone YF5031 assayed by MIP-1β activation with graded concentrations of the peptides LLWNGPMAV or KQWNGFIPV (mean and SEM).
