# Supplementary material for: T cell receptor alpha variable 12‐2 bias in the immunodominant response to Yellow fever virus
Source: Eur J Immunol. 2017 Dec 11;48(2):258–72. doi: 10.1002/eji.201747082 (PMC5887915; doi:10.1002/eji.201747082)
Supplement: Supplementary file 1 — Peer review correspondence [file EJI-48-258-s001.pdf]

European Journal of Immunology

T cell Receptor Alpha Variable 12-2 bias in the immunodominant response to Yellow fever Virus

Amandine Bovay, Vincent Zoete, Garry Dolton, Anna M. Bulek, David K. Cole, Pierre J. Rizkallah, Anna Fuller, Konrad Beck, Olivier Michielin, Daniel E. Speiser, Andrew K. Sewell, Silvia A. Fuertes Marraco

Marraco[1]Correspondence: Dr. Silvia A. Fuertes Marraco, Department of Oncology and Ludwig Cancer Research, Lausanne University Hospital (CHUV), Epalinges, Switzerland

---

Review Timeline:	Submission date:	13-Apr-2017
	First Editorial decision:	23-May-2017
	Revision received:	15-Aug-2017
	Accepted:	25-Sept-2017

Handling Executive Committee member: Prof. Annette Oxenius

Please note that the correspondence below does not include the standard editorial instructions regarding preparation and submission of revised manuscripts, only the scientific revisions requested and addressed.

First Editorial Decision
23-May-2017

Dear Dr. Fuertes Marraco,

Manuscript ID eji.201747082 entitled "High frequency and prevalence of Yellow Fever virus-specific CD8 T cells can be inherited" which you submitted to the European Journal of Immunology has been reviewed. The comments of the referees are included at the bottom of this letter.

You will see that a common concern of the referees is that the results are not based on data derived from crystal structures but rather in silico data, and that the limited functional data do not support your title/conclusion of heritability. Although referee 2 felt that the advance presented in your submission is incremental and justified rejection, we refer you to the guidance of Referee 1, who feels that:

European Journal of Immunology

"Overall, the authors interpret both the alanine scan and the CPL functional experiments to suggest â€œthat this TCR makes the majority of its critical contacts in this regionâ€. It is important that the functional data strictly speaking do not identify contact residues: the functionally determined effects could be explained both as a consequence of altering contact residues or as a consequence of altering framework determinants important for the overall conformation needed to properly present other residues being the true contact residues. In this context, it is surprising that the authors have not extended these functional experiments to test one of the TRAV12-2 negative A2/LLW-specific clones. Will the results of this experiment support the notion that TRAV12-2 is necessary to mediate interaction with the N-terminal peptide residues? This reviewer fear that this will not be the case, and that the entire line of reasoning behind these functional validation experiments may dissipate."

The Executive Editor strongly encourage you, based on the lack of experimental structural data and based on potential misunderstanding of the notion that â€œCD8 T-cell frequency and prevalence is heritableâ€, to tone down your conclusions (hence modify the title/abstract and discussion) accordingly.

A revised version of your manuscript that takes into account the comments of the referees, using those of Referee 1 as guidance, will be reconsidered for publication. Should you disagree with any of the refereesâ€™ concerns, you should address this in your point-by-point response and provide solid scientific reasons for why you will not make the requested changes.

You should also pay close attention to the editorial comments included below. *In particular, please edit your figure legends to follow Journal standards as outlined in the editorial comments. Failure to do this will result in delays in the re-review process.*

Please note that submitting a revision of your manuscript does not guarantee eventual acceptance, and that your revision will be re-reviewed by the referee(s) before a decision is rendered.

If the revision of the paper is expected to take more than three months, please inform the editorial office. Revisions taking longer than six months may be assessed by new referee(s) to ensure the relevance and timeliness of the data.

Once again, thank you for submitting your manuscript to European Journal of Immunology and we look forward to receiving your revision.

Yours sincerely,
Karen Chu

European Journal of Immunology

On behalf of Prof. Annette Oxenius

Dr. Karen Chu
Editorial Office
European Journal of Immunology
e-mail: ejied@wiley.com
www.eji-journal.eu

****

Reviewer: 1

Comments to the Author

The Yellow Fever vaccine, YF-17D, induces a strong and brisk immune response. Others and the authors have identified a particularly frequent and prevalent (i.e. immunodominant) CD8+ T cell response restricted by HLA-A*02:01 and specific for a NS4b 214-222 epitope, LLWNGPMAV (this cognate target is abbreviated A2/LLW). A2/LLW-specific CD8+ T cells could not only be detected in YF-17D vaccinated, but also in many unvaccinated (i.e. naÃ¯ve), HLA-A*02:01+ individuals. Here, the authors have demonstrated that A2/LLW-specific CD8+ T cells express a highly-skewed T cell receptor (TcR) repertoire with a strong bias (up to 80%) for the TcR alpha chain, TRAV12-2, both in YD-17D vaccinated and unvaccinated individuals. To understand this bias, the authors studied the functional characteristics of a panel of A2/LLW-specific CD8+ T cell clones. On a per cell basis, they found no functional difference with respect to cytotoxicity and secretion of a selected panel of cytokines, and no difference in the stability of TcR-A2/LLW interactions irrespective of whether the clonotypic TcR was TRAV12-2 positive, or not. The authors addressed the specificity of a TRAV12-2+, A2/LLW-specific T cell clone, YF5048, using combinatorial peptide libraries and alanine scans of the LLWNGPMAV epitope showing the crucial importance of the asparagine residue in position 4. The authors also studied the structural characteristics of the TRAV12-2+ TcR recognition of the cognate A2/LLW-epitope. They successfully generated a crystal structure of the A2/LWW complex, but unfortunately, they failed to generate a crystal structure of the YF5048 TcR in complex with A2/LLW. Instead, they developed an in silico model of this interaction. This model suggested that the TcR interaction with the cognate A2/LLW complex was dominated by the CDR1a loop positioned over the peptide N-terminal and contacting the asparagine residue in position 4 of the epitope. The CDR1 is encoded by the TRAV12-2 segment. The authors note similarities with other TRAV12-2+ T cell responses where CDR1 appear involved in peptide recognition. Since the CDR1 is

European Journal of Immunology

encoded in germline configuration, the authors argue that â€œthis supports the notion that, for certain CD8 T cell specificities, the human genome encodes TCR segments that are key for antigen binding, consequently favoring thymic output and allowing high frequency and prevalence, rendering these two properties heritable traitsâ€□.

This reviewer wonders whether it is doing this manuscript a great and misleading disservice when it stresses the concept that high frequency and prevalence) of certain CD8 T cell specificities are heritable traits, and when they do it to the extent that it enters into the paper title thereby becoming the take-home message. The reader may easily misconstrue this to mean that immunodominant CD8+ T cell responses are heritable; however, the all-important specificity of the response is not inherited. Even in this case, the specificity of the TcR is the result of a non-inheritable rearrangement as suggested by the authors themselves when they examine the responses of two different TRAV12-2+ T cell specificities and find no cross-reaction. This reviewer wonders whether it wouldnâ€™t be more appropriate and rewarding to stress the mechanistic concept that the germline encoded CDR1 can contribute significantly to the recognition of certain cognate peptide/MHC complexes and that this in some cases may explain immunodominance as well as the pre-existence of measurable T cell specificities even in naÃ¯ve individuals. It may also provide a fascinating explanation of the phenomenon of â€œpublic TcRsâ€□.

The authors admirably pursue a structural explanation of the TRAV12-2 bias. It is obvious that they would have been thrilled to report a crystal structure of the YF5048 TcR interaction with the cognate A2/LLW ligand, and it is understandable that they in lieu of structural data resort to an in silico model based on the known crystal structure the TcR of A2/ELA specific CD8+ T cell, MEL5, which represents another example of a TRAV12-2 bias. It is perhaps not surprising that the modeled YF5048 TRAV12-2 encoded structure resembles that of MEL5?

To support this model, the authors perform several functional analyses of the fine specificity of the TRAV12-2 positive YF5048 CD8+ T cell clone. To identify the important residues of the LLW peptide ligand they use an alanine scan and a more complicated combinatorial peptide library approach. The alanine scan shows that an alanine substitution in position 4 completely abrogates YF5048 reactivity suggesting that the asparagine in position 4 is an important, if not the most important, residue. The combinatorial peptide library (CPL) analysis is a much more detailed analysis; however, as it stands the results appears much less clear. First and foremost, the construction of the CPL is so superficially described that it compromises the interpretation of the CPL experiments (see below). Secondly, the results are interpreted to suggest that the â€œnumber of amino acid combinations that were recognized by the YF5048 TCR was particularly restricted within the central region of the peptide (residues 3-5)â€□. This reviewer does not find that the results support this interpretation. Of the TcR facing residues, positions 1, 7 and 9 appear just as discriminative as positions 4 and 5. It should also be noted that position

European Journal of Immunology

4 accepts a non-conservation substitution (N->R), yet obtain a heteroclitic improvement; a somewhat surprising effect if this is a contact residue. Overall, the authors interpret both the alanine scan and the CPL functional experiments to suggest â€œthat this TCR makes the majority of its critical contacts in this regionâ€□. It is important that the functional data strictly speaking do not identify contact residues: the functionally determined effects could be explained both as a consequence of altering contact residues or as a consequence of altering framework determinants important for the overall conformation needed to properly present other residues being the true contact residues. In this context, it is surprising that the authors have not extended these functional experiments to test one of the TRAV12-2 negative A2/LLW-specific clones. Will the results of this experiment support the notion that TRAV12-2 is necessary to mediate interaction with the N-terminal peptide residues? This reviewer fear that this will not be the case, and that the entire line of reasoning behind these functional validation experiments may dissipate.

It is stated in the abstract and elsewhere â€œthat TRAV12-2 does not confer a functional advantageâ€□. This is a quite broad and non-specific description. It is important to distinguish between the overall response and the per cell response. What the authors mean is that there is no difference between TRAV12-2 positive and negative CD8+ T cells on a per cell basis. This qualification needs to be spelled out. The increase in number (i.e. frequency) of responding T cells does translate into a functional advantage of a response including TRAV12-2 CD8+ T cells.

A more general criticism concerns a lack of attention to details, which compromises a proper understanding of the manuscript, a proper evaluation of the experimental results, and of attempts to repeat the experiments:

A) The description of the combinatorial peptide library (CPL) is insufficient. This reviewer interprets the CPL peptide to be a classical positional scanning combinatorial peptide library (PSCPL) where multiple sublibraries are made, each keeping one amino acid in one position fixed while all other positions have incorporated a mixture of the 20 naturally occurring amino acids (minus Cys). This would lead to the generation of 9 (positions) times 20 (naturally occurring amino acids â€“ now including Cys) = 180 sublibraries. This appear to be what Figure 7 seems to indicate. Now, each of these sublibraries contain 19^8 different peptides. This does not at all fit with the claim made in the methods section that â€œthe nonamer CPL contained a total of 4.8Ã—10^11 ((9+19)Ã—19^8) different nonamer peptides. In all likelihood the formulate should have read ((9x20)Ã—19^8) = 3e12; however, these would not all be different (some of the peptide sequences of one sublibrary would also be present in another sublibrary) and the real diversity covered would be a sequence space of 20^9 = 5.12x10^11 different peptides. None of these number are really that important, but it illustrates the important criticism that the description is not sufficiently detailed to allow a full understanding of the experiments performed; something that compromises interpretations.

B) The description of the production of recombinant HLA molecule is insufficient. It goes straight from

European Journal of
Immunology

inclusion bodies to folding complexes (P 20, line 420). Obviously, something happens in between, but what? The previous sentence on the expression references two papers by Garboczi et al from the 1990â€™es. One could assume that these references also covers the missing information, but do the authors mean that they are using methods developed 20 years ago without any modification? At face value, it would be guesswork and make it difficult to try to repeat these procedures.
C) page 3, line 32: â€œit is not unusual for the germline CDR1Î± loop to also contact peptide residues, and in some cases to dominate the contact with the peptide (4â€"6)â€□ where reference 4 is a Garboczi paper on recombinant production of peptide-MHC complexes with no apparent information pertaining the CDR1. What is the relevance of this reference?
D) page 6, lines 83-87 states: â€œWe next investigated this TRAV12-2 enrichment in A2/LLW-specific CD8 T cells from eight YF-17D vaccinees at the protein level (missing figure reference). We also compared A2/LLW-specific CD8 T â€¦ observed TRAV12-2 bias (missing figure reference)â€□. A little bit more detail and specific description would help in understanding what is meant by â€œprotein levelâ€□ and â€œcomparisonâ€□. Instead, the lack of figure references increases the sense of having to guess what the authors mean. This also true for line 93 where it is said â€œIn accordance with our previous studyâ€□ where a crucial reference is missing.

The manuscript could also have been improved with a little extra attention to fact checking and copy editing:
p18 line 375 HLA-A*0201/LLWNGPMAV (NS2b214-222). This is incorrect; the source protein is not a NS2b, rather it is NS4b.
p 10, line 178: â€œTyr8 of Tax in P(Î© square, square square;â€□ where there are obvious transliteration problems with square symbols inserted and a missing parenthesis end bracket. This is seen several places including in the references p30, line 635.
p16 line 320 â€" missing period in â€œvaccination In agreementâ€□ â€¨
P22 line 463ff â€" PMID numbers appears, which should have been removed in finalizing the manuscript
P25, line 505ff â€" formatting error for capital letter abbreviation of authors
P25, line 512-3 â€" garbled title and missing reference

Reviewer: 2

Comments to the Author
The work by Bovay et al. seeks to understand the preferential role of the TCR Va12-2 gene in TCRs that respond to a yellow fever epitope presented by HLA-A*0201. The authors compare this to the well-known case of TCRs that respond to the MART-1 epitope which also preferentially utilise Va12-2. The "meat" of

the story is that the germline CDR1alpha loop is predicted to form crucial interactions with the N-terminal half of the peptide, as seen with some MART-1/Melan-A TCRs and other Va12-2 TCRs that recognise viral antigens. This all makes good sense and is probably correct.

However the authors choose to discuss the CDR1alpha contribution in isolation - they do not discuss the fact that hypervariable loops will work together with CDR1alpha in determining specificity, as well as possibly CDR1beta across the interface and maybe even the CDR2 loops. The implication, intended or not, is that CDR1alpha is THE primary specificity driver. This is seen with the flawed title - reactivity can be inherited. Taken to its conclusion ALL T-cell reactivity can be inherited as all TCRs require some germline loops, constant domains, etc. in order to work. Therefore although the general importance of the CDR1alpha loop and 12-2 is probably correct as the authors surmise, the manuscript needs a concrete analysis of how the loop works with the others to determine specificity [or, a demonstration through mutational studies that the other loops can be greatly perturbed and reactivity maintained]. This will probably require a crystal structure of the complex and subsequent interrogation - the authors understand this and have tried to get this but have not been successful. Unfortunately the modelling performed in its absence is not going to report on what is really needed.

As it stands the work is incremental only and does not advance beyond what we already know: CDR1 loops, and the 1alpha loops of 12-2 genes in particular, can greatly contribute to antigen specificity.

****

First Revision – authors' response
15-Aug-2017

Point-by-point Reply to the Reviewer's comments

REVIEWER 1

The Yellow Fever vaccine, YF-17D, induces a strong and brisk immune response. Others and the authors have identified a particularly frequent and prevalent (i.e. immunodominant) CD8+ T cell response restricted by HLA-A*02:01 and specific for a NS4b 214-222 epitope, LLWNGPMAV (this cognate target is abbreviated A2/LLW). A2/LLW-specific CD8+ T cells could not only be detected in YF-17D vaccinated, but also in many unvaccinated (i.e. naïve), HLA-A*02:01+ individuals. Here, the authors have demonstrated that A2/LLW-specific CD8+ T cells express a highly-skewed T cell receptor (TcR) repertoire with a strong bias (up to 80%) for the TcR alpha chain, TRAV12-2, both in YD-17D vaccinated and unvaccinated individuals. To understand this bias, the authors studied the functional characteristics of a panel of A2/LLW-specific CD8+ T cell clones. On a per cell basis, they found no functional difference with respect to cytotoxicity and secretion of a selected panel of cytokines, and no difference in the stability of TcR-A2/LLW interactions irrespective of whether the clonotypic TcR was TRAV12-2 positive, or not. The authors addressed the specificity of a TRAV12-2+, A2/LLW-specific T cell clone, YF5048, using

combinatorial peptide libraries and alanine scans of the LLWNGPMAV epitope showing the crucial importance of the asparagine residue in position 4. The authors also studied the structural characteristics of the TRAV12-2+ TcR recognition of the cognate A2/LLW-epitope. They successfully generated a crystal structure of the A2/LWW complex, but unfortunately, they failed to generate a crystal structure of the YF5048 TcR in complex with A2/LLW. Instead, they developed an in silico model of this interaction. This model suggested that the TcR interaction with the cognate A2/LLW complex was dominated by the CDR1a loop positioned over the peptide N-terminal and contacting the asparagine residue in position 4 of the epitope. The CDR1 is encoded by the TRAV12-2 segment. The authors note similarities with other TRAV12-2+ T cell responses where CDR1 appear involved in peptide recognition. Since the CDR1 is encoded in germline configuration, the authors argue that "this supports the notion that, for certain CD8 T cell specificities, the human genome encodes TCR segments that are key for antigen binding, consequently favoring thymic output and allowing high frequency and prevalence, rendering these two properties heritable traits".

This reviewer wonders whether it is doing this manuscript a great and misleading disservice when it stresses the concept that high frequency and prevalence) of certain CD8 T cell specificities are heritable traits, and when they do it to the extent that it enters into the paper title thereby becoming the take-home message. The reader may easily misconstrue this to mean that immunodominant CD8+ T cell responses are heritable; however, the all-important specificity of the response is not inherited. Even in this case, the specificity of the TcR is the result of a non-inheritable rearrangement as suggested by the authors themselves when they examine the responses of two different TRAV12-2+ T cell specificities and find no cross-reaction. This reviewer wonders whether it wouldn't be more appropriate and rewarding to stress the mechanistic concept that the germline encoded CDR1 can contribute significantly to the recognition of certain cognate peptide/MHC complexes and that this in some cases may explain immunodominance as well as the pre-existence of measurable T cell specificities even in naïve individuals. It may also provide a fascinating explanation of the phenomenon of "public TcRs".

We thank Reviewer 1 for the detailed recapitulation and synthesis of the major points in our study. We acknowledge the concern raised by the reviewer regarding the possibility that the readership may have drawn misleading conclusions from our original title and thank them for their constructive feedback and suggestions for improvements. Reviewer 2 also raised concern about our choice of title. We have now changed the title of our manuscript to "T cell Receptor Alpha Variable 12-2 bias in the immunodominant HLA A2-restricted response to Yellow Fever Virus" in line with the suggestion from Reviewer 1. In our revised manuscript we have further clarified that the contribution of a germline-encoded gene segment for antigen recognition that we observed in the immunodominant A2/LLW-specific TRAV12-2+ TCRs does not apply to *all* immunodominant T cell responses (lines 317-320). To date, this mode of binding and TRAV skewing, has only been observed with A2/Melan A, A2/Tax9 and the A2/LLW Yellow Fever epitope studied here; it is not generally applicable. The germline-encoded CDR1α is likely to be a major contributor to antigen recognition by TRAV12-2+ A2/LLW-specific TCRs. However, the TRAV12-2 chain clearly cooperates with somatically rearranged CDR3s to determine antigen specificity as confirmed by the data we present on the lack of cross-reactivity between TRAV12-2+ A2/ELA-specific and A2/LLW-specific T cell clones. We have further stressed this point in lines 322-327.

The authors admirably pursue a structural explanation of the TRAV12-2 bias. It is obvious that they would have been thrilled to report a crystal structure of the YF5048 TcR interaction with the cognate A2/LLW ligand, and it is understandable that they in lieu of structural data resort to an in silico model based on the known crystal structure the TcR of A2/ELA specific CD8+ T cell, MEL5, which represents another example of a TRAV12-2 bias. It is perhaps not surprising that the modeled YF5048 TRAV12-2 encoded structure resembles that of MEL5?

We thank Reviewer 1 for their comprehension of our reasons for pursuing an in silico strategy to provide an explanation for the observed TRAV12-2 bias in this system. We were very disappointed not to succeed in generating an A2/LLW:TCR complex structure. This deficiency was certainly not due to a lack of effort on our part as we tried to refold seven different A2/LLW-specific TRAV12-2 TCRs. The MEL5 TCR was the obvious choice for modeling as the MEL5 alpha chain differs from that of YF5048 by just 3 residues in the CDR3; the remaining sequence including germline CDR1α and CDR2α is (logically) identical between the two TCRs. We have detailed this information in a new Fig S1A. In addition, the crystal structure of the A2/LLW pMHC complex that we solved shows that the MHC structure is nearly identical to the MHC structure present in the MEL5 TCR:pMHC complex, with a root mean square deviation of only 0.78 Å. The LLW peptide in the crystal structure that we solved has a very similar conformation to the ELA peptide in the MEL5:pMHC crystal – we have added this peptide superimposition as a new Fig S1B. Thus, we agree with the prediction that the modeling of YF5048 on MEL5 concludes that both have a very similar structure. The near identical sequence of YF5048 and MEL5 TCR alpha chains and the high similarity between the pMHC 3D structures of A2/LLW and A2/ELA suggest that this modeling has a high probability of being accurate. The observed similarities in 3D structures of the pMHCs and the sequence similarity between the TRAV chains support our use of homology modeling.

To support this model, the authors perform several functional analyses of the fine specificity of the TRAV12-2 positive YF5048 CD8+ T cell clone. To identify the important residues of the LLW peptide ligand they use an alanine scan and a more complicated combinatorial peptide library approach. The alanine scan shows that an alanine substitution in position 4 completely abrogates YF5048 reactivity suggesting that the asparagine in position 4 is an important, if not the most important, residue. The combinatorial peptide library (CPL) analysis is a much more detailed analysis; however, as it stands the results appears much less clear. First and foremost, the construction of the CPL is so superficially described that it compromises the interpretation of the CPL experiments (see below). Secondly, the results are interpreted to suggest that the "number of amino acid combinations that were recognized by the YF5048 TCR was particularly restricted within the central region of the peptide (residues 3-5)". This reviewer does not find that the results support this interpretation. Of the TcR facing residues, positions 1, 7 and 9 appear just as discriminative as positions 4 and 5.

- We acknowledge the concerns from Reviewer 1 regarding the CPL assay. We shortened this section considerably in our original manuscript so that we could conform to the very tight word limit. We apologize if this negatively impacted the clarity of this section. We have re-written the results section for the alanine scan and CPL experiments (lines 204-240). We now start with the alanine scans and then move on to the CPL assays as we feel that this allows the data to be explained more concisely (Fig 7A and B). Indeed, the index residues of the peptide are well represented at all but 2 positions (7 and 8) of the CPL assay and therefore have now been described accordingly.

It should also be noted that position 4 accepts a non-conservation substitution (N->R), yet obtain a heteroclitic improvement; a somewhat surprising effect if this is a contact residue.

We thank Reviewer 1 for pinpointing this observation on the Asn4→Arg4 substitution. We actually tested stimulating A2/LLW-specific clones with the peptide containing this single substitution (LLWRGPMAV) and observed a complete abrogation of the response, similarly to the mutation N4→A4 (Fig 7A). This again supports the importance of the position 4 of the peptide. This could be explained by the fact that an Arg at position 4 only works with Gly at position 3, for instance. An Arg at position 4 might disrupt the interaction when there is a Trp at position

European Journal of Immunology

3. We have added this control experiment in Fig S3A and B in our revised manuscript. Large bulky amino acids at position 3 in HLA A2-bound peptides can form a bridge with more C-terminal peptide residues (usually position 5; *J Biol Chem* 2016, **291**, 8951-8959). Replacing residue 3 with a smaller side chains such as alanine or glycine can abolish this important intra-peptide stabilization and have knock-on effects at peptide residue 4 that impinge on TCR binding. We have similar findings in other systems (in review at *J Exp Med* and unpublished). Overall, these results point towards a common mechanism of peptide presentation by HLA A2 in which the residue at position 3 can play a pivotal role in maintaining antigenic identity. Changing the amino acid at position 3 can allow accommodation of different residues at position 4 in other systems we have studied. Unfortunately, such suggestions remain conjecture with A2/LLW in the absence of TCR binding data. Importantly, CPL data should not entirely be interpreted in the context of the wildtype peptide backbone. We have examples of strong agonists with other TCRs where all amino acids in the peptide are different. Peptide recognition is all about the *context*, and CPL-screening allows every context. Interestingly, for the second TRAV12-2+ clone that we examined by CPL, we found an agonist peptide that preserved only the central index residues 3-5 and the second anchor position (KQWNGPFIPV) and induced a response superior to the WT peptide (this is now shown in Fig S3C).

Overall, the authors interpret both the alanine scan and the CPL functional experiments to suggest "that this TCR makes the majority of its critical contacts in this region". It is important that the functional data strictly speaking do not identify contact residues: the functionally determined effects could be explained both as a consequence of altering contact residues or as a consequence of altering framework determinants important for the overall conformation needed to properly present other residues being the true contact residues. In this context, it is surprising that the authors have not extended these functional experiments to test one of the TRAV12-2 negative A2/LLW-specific clones. Will the results of this experiment support the notion that TRAV12-2 is necessary to mediate interaction with the N-terminal peptide residues? This reviewer fear that this will not be the case, and that the entire line of reasoning behind these functional validation experiments may dissipate.

- We thank Reviewer 1 for the pertinent question on whether the CPL using a TRAV12-2 negative TCR would show whether the central region of the peptide is crucial. We considered this point and now show the CPL results for two TRAV12-2 negative clones as well as another TRAV12-2 positive clone (Fig S2). The CPLs for the TRAV12-2 positive clones are similar, both clones preferring the index residue sub-libraries at the central positions (positions 3 to 5) of the peptide. The TRAV12-2 negative clone YF5001 was more degenerate at positions 3, 4 and 5, with multiple non-index residues also being recognized. TRAV12-2 negative clone YF5048NN1 showed a preference to index residues at positions 3 and 5, but no recognition for Asn4 and instead preferred Ser4.. We hope that these data reassure Reviewer 1 and further support the importance of the central region of the peptide LLW in the binding of TRAV12-2 positive TCRs.

It is stated in the abstract and elsewhere "that TRAV12-2 does not confer a functional advantage". This is a quite broad and non-specific description. It is important to distinguish between the overall response and the per cell response. What the authors mean is that there is no difference between TRAV12-2 positive and negative CD8+ T cells on a per cell basis. This qualification needs to be spelled out. The increase in number (i.e. frequency) of responding T cells does translate into a functional advantage of a response including TRAV12-2 CD8+ T cells.

European Journal of Immunology

We fully agree on the wording suggested by Reviewer 1 and thank the reviewer for this clarification. It is indeed important to point out the difference between the functional effect of TRAV12-2 at the clonal and the population levels. In fact, we do not see any difference of functionality using CD8 T cell clones (on a "per cell basis") but we strongly believe that TRAV12-2 expression has a functional advantage for the organism, at the population level, by increasing the frequency in the naïve compartment. To further emphasize this aspect, we have made changes in the section title (line 123) and lines 277-279

A more general criticism concerns a lack of attention to details, which compromises a proper understanding of the manuscript, a proper evaluation of the experimental results, and of attempts to repeat the experiments:
A) The description of the combinatorial peptide library (CPL) is insufficient. This reviewer interprets the CPL peptide to be a classical positional scanning combinatorial peptide library (PSCPL) where multiple sublibraries are made, each keeping one amino acid in one position fixed while all other positions have incorporated a mixture of the 20 naturally occurring amino acids (**minus Cys**). This would lead to the generation of 9 (positions) times 20 (naturally occurring amino acids – now including Cys) = 180 sublibraries. This appears to be what Figure 7B seems to indicate. Now, each of these sublibraries contain 19^8 different peptides.

- Reviewer 1 is absolutely correct; especially highlighting the fact that cysteine is absent from the degenerate positions.

This does not at all fit with the claim made in the methods section that "the nonamer CPL contained a total of 4.8×10^11 ((9+19)×19^8) different nonamer peptides. In all likelihood the formulate should have read ((9x20)×19^8) = 3e12; however, these would not all be different (some of the peptide sequences of one sublibrary would also be present in another sublibrary) and the real diversity covered would be a sequence space of 20^9 = 5.12x10^11 different peptides.

- Since cysteine is absent from the degenerate positions, none of the peptide possibilities containing 2 or more cysteines are included in the library. This covers not only those peptides containing 2 Cys residues but also 9 Cys residues or 8 Cys residues etc. Thus, it means 20^9 minus all these possibilities = 4.8×10^11 ((9+19)×19^8).

None of these number are really that important, but it illustrates the important criticism that the description is not sufficiently detailed to allow a full understanding of the experiments performed; something that compromises interpretations.

- As previously mentioned, we were strongly restricted in the description of the methods because of the word count limitation. We concede that this is important information for both the comprehension of the assay and to allow reproducibility. We have extended the description and explanation of the CPL assay as much as possible while giving the appropriate references for further details (refs 37 and 38 in the manuscript).

B) The description of the production of recombinant HLA molecule is insufficient. It goes straight from inclusion bodies to folding complexes (P 20, line 420). Obviously, something happens in between, but what? The previous sentence on the expression references two papers by Garboczi et al from the 1990'es. One could assume that these references also covers the missing information, but do the authors mean that they are using methods developed 20

years ago without any modification? At face value, it would be guesswork and make it difficult to try to repeat these procedures.

- Again, we had to keep our methods short. We have now added a new reference, which describes in detail, including video support, all the steps of expression, refold and purification (ref 39 in the manuscript).

C) page 3, line 32: “it is not unusual for the germline CDR1α loop to also contact peptide residues, and in some cases to dominate the contact with the peptide (4–6)” where reference 4 is a Garboczi paper on recombinant production of peptide-MHC complexes with no apparent information pertaining the CDR1. What is the relevance of this reference?

- We apologize for this lack of attention to detail and thank the reviewer for their careful reading of our manuscript. We have now corrected the reference for Garboczi et al 1996 (Nature) instead of Garboczi et al 1992 (PNAS) (ref 4 in the manuscript).

D) page 6, lines 83-87 states: “We next investigated this TRAV12-2 enrichment in A2/LLW-specific CD8 T cells from eight YF-17D vaccinees at the protein level (missing figure reference). We also compared A2/LLW-specific CD8 T … observed TRAV12-2 bias (missing figure reference)”. A little bit more detail and specific description would help in understanding what is meant by “protein level” and “comparison”. Instead, the lack of figure references increases the sense of having to guess what the authors mean. This also true for line 93 where it is said “In accordance with our previous study” where a crucial reference is missing.

- We apologize for this oversight. The figure references are now included in the text.

The manuscript could also have been improved with a little extra attention to fact checking and copy editing:
p18 line 375 HLA-A*0201/LLWNGPMAV (NS2b214-222). This is incorrect; the source protein is not a NS2b, rather it is NS4b.
p 10, line 178: “Tyr8 of Tax in P(Ω square, square square;” where there are obvious transliteration problems with square symbols inserted and a missing parenthesis end bracket. This is seen several places including in the references p30, line 635.
p16 line 320 – missing period in “vaccination In agreement”

P22 line 463ff – PMID numbers appears, which should have been removed in finalizing the manuscript
P25, line 505ff – formatting error for capital letter abbreviation of authors
P25, line 512-3 – garbled title and missing reference

- We apologize for the editing mistakes, which have now been corrected. We thank Reviewer 1 for the detailed revision of our text.

European Journal of Immunology

REVIEWER 2

The work by Bovay et al. seeks to understand the preferential role of the TCR Va12-2 gene in TCRs that respond to a yellow fever epitope presented by HLA-A*0201. The authors compare this to the well-known case of TCRs that respond to the MART-1 epitope which also preferentially utilise Va12-2. The "meat" of the story is that the germline CDR1alpha loop is predicted to form crucial interactions with the N-terminal half of the peptide, as seen with some MART-1/Melan-A TCRs and other Va12-2 TCRs that recognise viral antigens. This all makes good sense and is probably correct.

- We are pleased that Reviewer 2 agrees with our predictions.

However the authors choose to discuss the CDR1alpha contribution in isolation - they do not discuss the fact that hypervariable loops will work together with CDR1alpha in determining specificity, as well as possibly CDR1beta across the interface and maybe even the CDR2 loops. The implication, intended or not, is that CDR1alpha is THE primary specificity driver. This is seen with the flawed title - reactivity can be inherited. Taken to its conclusion ALL T-cell reactivity can be inherited as all TCRs require some germline loops, constant domains, etc. in order to work. Therefore although the general importance of the CDR1alpha loop and 12-2 is probably correct as the authors surmise, the manuscript needs a concrete analysis of how the loop works with the others to determine specificity [or, a demonstration through mutational studies that the other loops can be greatly perturbed and reactivity maintained]. This will probably require a crystal structure of the complex and subsequent interrogation - the authors understand this and have tried to get this but have not been successful. Unfortunately the modelling performed in its absence is not going to report on what is really needed.

- This point is also raised by Reviewer 1 - please see our answers above (page 1).

As it stands the work is incremental only and does not advance beyond what we already know: CDR1 loops, and the 1alpha loops of 12-2 genes in particular, can greatly contribute to antigen specificity.

To date there are only two examples of this binding mechanism in the literature (MEL5 and A6 TCRs). Both of these examples are for antigens known to induce T-cell responses that are heavily enriched for TRAV12-2, although this skewing has not been widely reported for the A2/Tax9 peptide. Speculation remains as to whether this TRAV12-2 bias is an odd curio. The additional example we provide in our study with a completely different peptide increases the likelihood that there might be other TRAV12-2 biased response to other epitopes. Our new data also open up the possibility that other responses might exist that are biased for other TCR variable gene elements as the result of germline encoded contacts. Furthermore, we now provide proof, for the first time, that there is no cross-reactivity between two TRAV12-2 positive TCR specificities that use the germline-encoded CDR1α to recognize their antigen (Fig 8). Given the above, we strongly disagree that our study does not advance knowledge beyond that already known.

Second Editorial Decision

14-Sep-2017

Dear Dr. Fuertes Marraco,

It is a pleasure to provisionally accept your manuscript entitled "T cell Receptor Alpha Variable 12-2 bias in the immunodominant response to Yellow fever Virus" for publication in the European Journal of Immunology. For final acceptance, please follow the instructions below and return the requested items as soon as possible as we cannot process your manuscript further until all items listed below are dealt with. Please note that EJI articles are now published online a few days after final acceptance (see Accepted Articles: http://onlinelibrary.wiley.com/journal/10.1002/(ISSN)1521-4141/accepted). The files used for the Accepted Articles are the final files and information supplied by you in Manuscript Central. You should therefore check that all the information (including author names) is correct as changes will NOT be permitted until the proofs stage.

We look forward to hearing from you and thank you for submitting your manuscript to the European Journal of Immunology.

Yours sincerely,
Marta Vuerich

on behalf of
Prof. Annette Oxenius

Dr. Marta Vuerich
Editorial Office
European Journal of Immunology
e-mail: ejied@wiley.com
www.eji-journal.eu
